# MicroRNA-132 provides neuroprotection for tauopathies via multiple signaling pathways

**DOI:** 10.1007/s00401-018-1880-5

**Published:** 2018-07-07

**Authors:** Rachid El Fatimy, Shaomin Li, Zhicheng Chen, Tasnim Mushannen, Sree Gongala, Zhiyun Wei, Darrick T. Balu, Rosalia Rabinovsky, Adam Cantlon, Abdallah Elkhal, Dennis J. Selkoe, Kai C. Sonntag, Dominic M. Walsh, Anna M. Krichevsky

**Affiliations:** 1 Department of Neurology, Ann Romney Center for Neurologic Diseases, Brigham and Women’s Hospital and Harvard Medical School, 60 Fenwood Rd, 9006, Boston, MA 02115 USA; 2 Department of Psychiatry, McLean Hospital and Harvard Medical School, Belmont, MA 02478 USA; 3 Division of Transplant Surgery and Transplantation Surgery Research Laboratory, Brigham and Women’s Hospital and Harvard Medical School, Boston, MA USA; 4 Harvard Medical School Initiative for RNA Medicine, Boston, MA 02115 USA

**Keywords:** Alzheimer’s disease, Tauopathies, Neurodegeneration, Neuroprotection, MicroRNA, Non-coding RNA

## Abstract

MicroRNAs (miRNA) regulate fundamental biological processes, including neuronal plasticity, stress response, and survival. Here, we describe a neuroprotective function of miR-132, the miRNA most significantly downregulated in neurons in Alzheimer’s disease. We demonstrate that miR-132 protects primary mouse and human wild-type neurons and more vulnerable Tau-mutant neurons against amyloid β-peptide (Aβ) and glutamate excitotoxicity. It lowers the levels of total, phosphorylated, acetylated, and cleaved forms of Tau implicated in tauopathies, promotes neurite elongation and branching, and reduces neuronal death. Similarly, miR-132 attenuates PHF-Tau pathology and neurodegeneration, and enhances long-term potentiation in the P301S Tau transgenic mice. The neuroprotective effects are mediated by direct regulation of the Tau modifiers acetyltransferase EP300, kinase GSK3β, RNA-binding protein Rbfox1, and proteases Calpain 2 and Caspases 3/7. These data suggest miR-132 as a master regulator of neuronal health and indicate that miR-132 supplementation could be of therapeutic benefit for the treatment of Tau-associated neurodegenerative disorders.

**Electronic supplementary material:**

The online version of this article (10.1007/s00401-018-1880-5) contains supplementary material, which is available to authorized users.

## Introduction

Alzheimer’s disease (AD) is a progressive neurodegenerative disorder typified by profound synaptic loss, brain atrophy, and the presence of extracellular plaques composed of amyloid β-protein (Aβ), and intracellular neurofibrillary tangles (NFTs) formed by hyperphosphorylated Tau [3, 27]. NFTs are also pathogenomic for a range of disorders in which Tau deposits occur in the absence of plaques. The major primary tauopathies include: frontotemporal dementia (FTD), progressive supranuclear palsy (PSP), Pick’s disease, and corticobasal degeneration. New therapeutic strategies and targets are desperately needed to treat these devastating diseases.

miRNAs are small regulatory molecules that post-transcriptionally repress gene expression and thereby regulate diverse biological processes, including neuronal differentiation, plasticity, survival, and regeneration [42]. miRNAs are often considered as determinants of cell fate and are also increasingly acknowledged as prime regulators involved in various brain pathologies ranging from neurodevelopmental disorders to brain tumors, to neurodegenerative diseases [26]. Given its immense complexity, the brain expresses the richest repertoire of miRNA species, with specific miRNAs being highly enriched in certain cell types of the brain, e.g., developing or mature cortical neurons. Early studies reported that deficiency of Dicer, the key ribonuclease in miRNA biogenesis, resulted in progressive miRNA loss, death of Purkinje neurons, and cerebellar degeneration [10, 54]. Several neuronal miRNAs have been directly linked to the regulation of key factors involved in AD, including APP and Aβ production and clearance [26]. Although multiple lines of evidence suggest that miRNAs may contribute to the progression of neurodegenerative diseases, the complexity of miRNA regulation in targeting many genes and pathways simultaneously raised concerns about their therapeutic utility as targetable molecules.

One of the most abundant brain-enriched miRNAs is miR-132, which plays a key role in both neuron morphogenesis and plasticity. miR-132, transcribed by the activity-dependent transcription factor CREB, modulates axon and dendrite development and spine maturation in response to a variety of signaling pathways [29, 33]. Deletion of the miR-132 locus decreases dendritic arborization, length, and spine density, impairs integration of newborn neurons, and reduces synapse formation in the adult hippocampus [33, 35, 65]. miR-132 inhibition induces apoptosis in cultured cortical and hippocampal primary neurons via PTEN/AKT/FOXO3 signaling [67]. Notably, miR-132-deficient mice exhibit Tau hyperphosphorylation, aggregation, and decreased memory—all of which are hallmarks of AD [53, 56]. Deletion of miR-132 also fosters Aβ production and plaque accumulation in a triple transgenic mouse AD model [22]. Importantly, multiple studies have shown that miR-132 is the most downregulated miRNA in postmortem AD brain with reductions in miR-132 occurring before neuronal loss and associated with progression of both amyloid and Tau pathology [22, 31, 45, 46, 53, 67].

We hypothesized that supplementation of miR-132 activity may protect against AD and other tauopathies. In support of this idea, we report results of a high-content miRNA screen performed on primary mouse and human neurons treated with either an AD-specific insult (Aβ) or excitotoxic levels of glutamate. Among the miRNAs expressed in the brain, miR-132 exhibits the strongest neuroprotective activity against both Aβ and glutamate. Furthermore, overexpression of miR-132 reduced phosphorylated, acetylated, and cleaved forms of Tau in primary neurons, as well as Tau pathology and caspase-3-dependent apoptosis in PS19 (TauP301S) mice. Functionally, miR-132 overexpression enhanced long-term potentiation (LTP) in WT mice and rescued the impairment of LTP seen in PS19 mice. These results suggest that miR-132 replacement could provide neuroprotection and therapeutic value for Tau-associated neurodegenerative disorders, including AD and FTD.

## Materials and methods

### Primary neuronal cultures and their analysis

Primary cortical and hippocampal neuron cultures were prepared from WT (E18) and PS19 (P1; JAX:008169) mice, and human fetal cortical specimens (provided by Advanced Bioscience Resources, Alameda, CA, USA). All studies have been approved and performed in accordance with Harvard Medical Area and BWH Standing Committee (IACUC) guidelines. Brain tissues were dissected, dissociated enzymatically by papain, and mechanically by trituration through Pasteur pipette, plated and cultured as previously described [64]. Imaging of the cultures was performed using the IncuCyteTM Live-Cell Imaging System (Essen BioScience). Cell confluency, cell body number, neurite length, and branching points were monitored and quantified using the IncuCyteTM software. Neuron viability was measured using the WST1 assay, following the manufacturer’s instructions (Roche).

### Transfections of primary neurons

Transfections of miRNA mimics (Miridian oligonucleotides at 20 nM final concentration, Dharmacon), inhibitors (LNA-containing, at 50 nM, Exiqon), siRNAs (at 25 nM, Dharmacon), and the corresponding control oligonucleotides of the same chemistries to primary mouse and human neurons were carried out using the NeuroMag technology (OZ Biosciences). The cultures were incubated with the transfection mixture in a standard incubator overnight. Half of the media was replaced next morning, and the remaining media were replaced at later time points. Transfection efficacy for miRNA inhibitors, mimics, and siRNA is 95–100% [67].

### SEC isolation of Aβ monomer and preparation of ½-*t*_max_ Aβ (1–42)

Based on the general consensus that aggregation of Aβ is required for toxicity, we employed a partially aggregated preparation of Aβ(1–42) that contained both amyloid fibrils and Aβ monomer [4, 62]. This preparation is referred to as 1/2*t*_max_, because it is produced by incubating Aβ monomer for a period that yields half of the maximal level of thioflavin T. When used at concentration ≥ 10 μM, 1/2*t*_max_ can cause the compromise and death of cultured rodent and human neurons within a period of a few days [4, 62]. Synthetic monomer Aβ (1–42) (human sequence) was obtained from rPeptide (A-1165-2). Briefly, Aβ (1–42) was dissolved at 1 mg/ml in 50 mM Tris–HCl, pH 8.5, containing 7 M guanidinium HCl, and 5 mM ethylenediamine tetraacetic acid, and incubated at room temperature overnight. The sample was then centrifuged at 16,000×*g* for 30 min and the upper 90% of supernatant applied to a Superdex 75 10/300 size exclusion column (GE Healthcare Biosciences), eluted at 0.5 ml/min with 50 mM ammonium bicarbonate, pH 8.5. Absorbance was monitored at 280 nm. Fractions of 0.5 ml were collected. Peak fractions were pooled and the concentration of Aβ determined using *ε*275 = 1361/M/cm. Thereafter, ½*t*_max_ Aβ(1–42) was prepared as described previously [40]. The samples were aliquoted, flash frozen on dry ice, and stored at − 80 °C.

### Real-time quantitative RT-PCR

Total RNA was extracted from cultures and tissues with Exiqon RNA isolation kit, according to the manufacturer’s instructions. For miRNA quantifications, TaqMan^®^ miRNA assays (Life Technologies) were used, and miRNA levels were normalized to the geometrical mean of the uniformly expressed miR-99a, miR-181a, and U6 snRNA. The mRNA levels were monitored by qRT-PCR with specific primers listed in the Supplemental Table S1, on the ViiA-7 System (Thermo Fisher Scientific). Threshold cycles (Cts) were generated automatically, and the relative expressions were shown as 2 ^−Δ*Ct*^ . mRNA levels were normalized to the geometrical mean of 18 rRNA, ACTB, and PABP2 mRNAs.

### Western blotting analysis

Proteins have been extracted and the concentrations determined by Pierce^™^ BCA Protein Assay Kit. For Western blot analysis, the proteins have been resolved on the SDS-PAGE, transferred to 0.45 μm nitrocellulose membranes (BioRad), blocked with 5% non-fat dry milk in PBS with 0.1% Tween 20, and processed for immunodetection. Sarkosyl-insoluble tau was isolated as previously described [5]. The following primary antibodies were used following the manufacturer’s instructions: Tau 5, Tau 46, Tau-PHF, Rbfox1, Calpain 2, cleaved Caspase-3, cleaved Caspase-7, GSK3β, EP300, and β-Actin (Cell Signaling). Anti-acetyl-Tau AC312 (rabbit anti-ac-K174 Tau) and MAB359 (rabbit anti-ac-K274 Tau) kindly provided by Li Gan’s laboratory were used at 1/5000 dilution. Antibody detection was performed with the HRP-coupled goat secondary anti-mouse or anti-rabbit antibodies (Immunoresearch), followed by the ECL reaction (Perkin Elmer) and exposure to Fuji X-ray films. The films were scanned and signals quantified using the ImageJ software.

### Detection of intracellular and extracellular Tau using ELISAs

Two ELISAs were used in this study. One which is similar to clinically approved assays which employ mid-region directed mAbs and are often erroneously referred to as total tau assays, and the other a novel C-terminal ELISA that uses mAbs specific for the C-terminus and MTBR domains of Tau. The mid-region tau ELISA was performed essentially as described previously [28]. The anti-tau monoclonal antibody BT2 (Thermo Scientific) at 2.5 μg/ml in TBS was used for capture and Tau5 conjugated to alkaline phosphatase was used for detection. Samples were analyzed in duplicate, whereas blanks and Tau441 standards (7.8–8000 pg/ml) were analyzed in triplicate. Standard curves were fitted to a five-parameter logistic function with 1/Y2 weighting, using MasterPlex ReaderFit (MiraiBio). The lower limit of quantification (LLOQ) was calculated for each plate and for the results shown the LLoQ was 31 pg/ml. The C-terminal ELISA was performed exactly as for the mid-region assays except the polyclonal antibody K9JA (243-441aa) was used for capture and the mAb TauAB (425-441aa) was used for detection. For the results shown, the LLoQ of the assay was 7.8 pg/ml.

### Cross-linking and immunoprecipitation (iCLIP)

iCLIP was performed according to the published protocol [25], with minor modifications. Briefly, mouse neurons were irradiated with UV-C light to covalently cross-link proteins to nucleic acids (400 J/m^2^). Upon cell lysis, samples were subjected to DNase treatment and RNA was partially fragmented using low concentrations of the RNase I (0.002 U/ml, 5 min), followed by the treatment with the RNase inhibitor (RNAsin Plus at 0.5 U/μl) to quench RNase activity. The Rbfox1–RNA complexes were immunopurified using the anti-Rbfox1 antibody (Cell Signaling) immobilized on immunoglobulin G-coated magnetic beads. RNA was isolated and precipitated, and the RT-PCR reactions performed with the Tau-specific primers to amplify different segments of the mRNA.

### Validation of miR-132 targets by luciferase reporter assay

Full-length 3′ UTR sequences of Gsk3β, Calpain2, and Rbfox1 were cloned into psiCHECK2 plasmid (Promega, C8021) downstream of *renilla* luciferase, using *Xho*I and *Not*I. Mutations in the miR-132 binding sites were introduced to these constructs using the QuikChange Multi Site-Directed Mutagenesis Kit (Stratagene). Primers used for cloning and mutagenesis are indicated in Table S1. Four hundred nanogram of the constructs were co-transfected with either miRNA mimics (25 nM final concentration) or LNA inhibitors (50 nM), in Lipofectamine 2000, to the SH-SY5Y cells grown in 96-well plates. Alternatively, for primary neurons, 1 µg of the constructs was used per well in 24-well plates. Two days after transfections, luciferase luminescence was measured using the Dual-Glo Luciferase Assay System (Promega, E2920) and detected with Infinite F200 plate reader (TECAN). Renilla luminescence was normalized with that of firefly and the signals were presented as renilla/firefly relative luminescence.

### Lentivirus production and stereotaxic brain injections

For lentivirus production, the miR-132-expressing PL13-pSyn-mmu-miR-132-IRES2-EGFP or control PL13-pSyn-IRES2-EGFP plasmid was co-transfected with packaging psPAX2 plasmids and VSV-G envelope-expressing plasmid (Addgene plasmids #12259 and #12260), and the viruses concentrated by additional ultracentrifugation at 25,000 rpm. Lentivirus titers were determined by PCR and functional titer was further determined by serial dilutions in 293T cells, using GFP fluorescence. The titer was estimated using the following formula: titer (TU/ml) = number of transduced cells in day 1 × percentage of GFP^+^ cells × 1000/volume of lentivirus used (ml). The lentivirus-expressing miR-132 (LV-miR132) or empty vector (EV) (2 μl) was stereotactically injected at 6 × 10^6^ TU/ml to the CA1 region of the right hippocampus (Bregma coordinates: 2.5 mm posterior, 1.7 mm lateral, and 1.8 mm ventral) P–A, 0.5 mm; C–L, 1.7 mm; D–V, 2.3 mm) of C57BL/6J and PS19 mice. The animals were randomized to the treatment and control groups. All animal studies have been approved and performed in accordance with Harvard Medical Area and BWH Standing Committee for Animal Care (IACUC) guidelines.

### Immunohistochemistry (IHC)

Mice were sacrificed by CO_2_ exposure following cervical dislocation, and the brains fixed in 4% paraformaldehyde, embedded, and cryo-sectioned. The 16-μm-thick sections were immunostained for NeuN, GFAP, Cleaved Caspase-3, and Tau-PFH with antibodies from Cell Signaling. The sections were first incubated in the blocking solution (7.5% NGS; 0.4% Triton; 1% BSA; PBS) for 2 h, followed by the overnight incubation in antibody-containing solution (5% NGS; 0.2% Triton; 0.5% BSA; PBS), and 2.5-h incubation with a secondary antibody (either AlexaFluor 568 or AlexaFluor 488; Invitrogen). IHC was visualized by Zeiss confocal microscopy at 20× magnification, and the images were processed with a computerized image analysis system (ZEN 2012 SP2 Software, Zeiss).

### Electrophysiology

Mouse brains were removed and submerged in ice-cold oxygenated cutting solution. Transverse slices (350-μm-thick) were cut with a vibroslicer from the middle portion of each hippocampus. Slices were incubated in artificial cerebrospinal fluid (ACSF), transferred to the recording chamber, and continuously perfused in ACSF saturated with 95% O_2_ and 5% CO_2_. Field excitatory postsynaptic potentials (fEPSP) were recorded in the CA1 region of the right hippocampus. Test responses were recorded for 20–30 min before the experiment. LTP was induced by two consecutive trains (1 s) of stimuli at 100 Hz separated by 20 s. The field potentials were amplified using Axon Instruments 200B amplifier and digitized with Digidata 1322A. Traces were obtained by pClamp 9.2 and analyzed using the Clampfit 9.2.

## Results

### A miRNA screen identifies miR-132 as strongly neuroprotective against Aβ and glutamate excitotoxicity in primary neurons

**Fig. 1:**
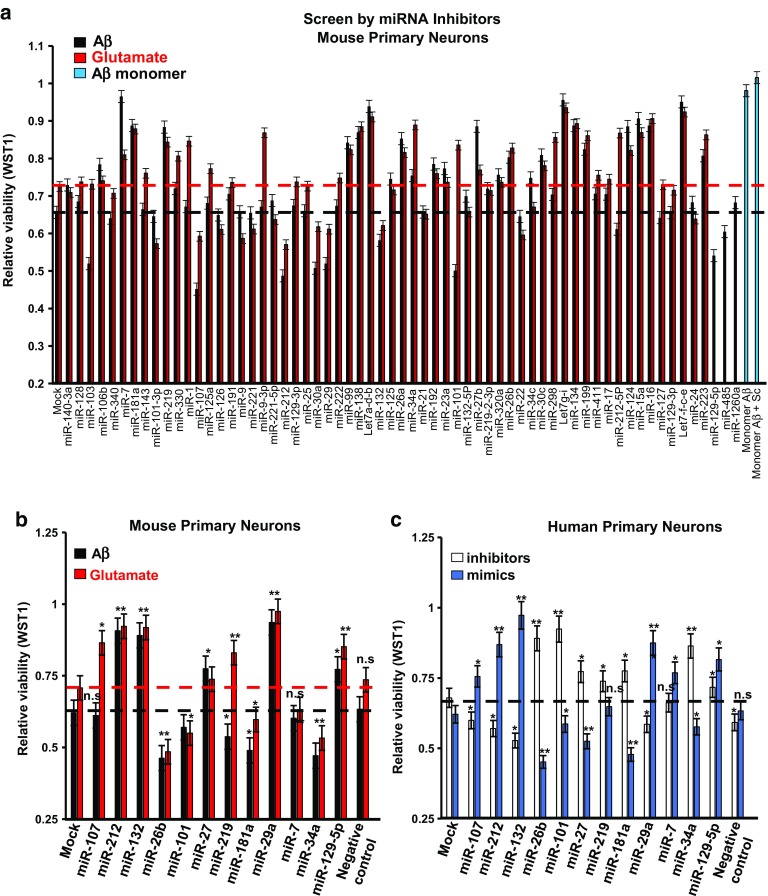
Screen in primary neurons identifies miRNAs protecting or exacerbating Aβ and glutamate toxicity. Primary neurons were transfected with either miRNA oligonucleotide inhibitors (anti-miRNA) or mimics (miRNA), and treated with Aβ (1, 5 µM), glutamate (100 µM) or vehicle. WST1 assays were performed 3 days later, and results normalized to transfected, vehicle-treated neurons (see also Figure S1). Six wells per condition were analyzed. **a** Relative viability of mouse hippocampal neurons transfected with the indicated sequence-specific anti-miRNAs. The value of “1” corresponds to the viability of untreated controls neurons. Treatment with non-toxic Aβ monomer does not reduce the viability (shown by two bars on the right side of the panel). The horizontal black and red lines mark the viability of untransfected neurons treated with Aβ or glutamate, respectively. **b** Relative viability of mouse hippocampal neurons transfected with the indicated miRNA mimics. **c** Relative viability of human primary cortical neurons pre-transfected with either miRNA inhibitors or mimics and then treated with Aβ. miR-132 mimic induced the most significant increase of neuronal survival in both mouse and human cultures (*P < 0.05 and **P < 0.01), *n* = 6, Student’s *t* test, control versus miR-132 inhibitor or mimic condition). Graphical data are shown as mean ± SEM

To identify endogenous miRNAs with neuroprotective properties, we performed a screen on 63 conserved most abundant neuronal miRNAs that together account for more than 90% of all miRNA expressed in the adult mouse brain [2]. Individual miRNAs were inhibited in mouse primary hippocampal and cortical neurons by specific locked nucleic acid (LNA)-based antisense oligonucleotide inhibitors (anti-miRNAs). Alternatively, cells were transfected with non-targeting control oligonucleotides of the same chemistry. The efficacy of anti-miRNAs was confirmed for selected miRNAs, using a panel of their previously validated mRNA targets [7] (Fig. S1a). The neurons were either transfected at DIV7 and exposed to glutamate (100 μM) 2 days later or transfected at DIV19 followed by the treatment with toxic Aβ (1, 5 μM) (Fig. S1b, c, d). Based on the general consensus that aggregation of Aβ is required for toxicity, we employed a partially aggregated preparation of Aβ (1–42) that contained both amyloid fibrils and Aβ monomer and is referred to as ½t_max_Aβ [4, 62]. Metabolic activity and cell viability were assessed by WST1 assays 3 days after glutamate or Aβ exposure, and the effects of miRNA inhibitors normalized to mock and control oligonucleotide (Fig. 1a). We observed that inhibition of specific miRNAs protected against glutamate (e.g., anti-let-7g, i), Aβ toxicity (e.g., anti-miRNA-7), or both toxic stimuli (e.g., let-7c, e) (Fig. 1a). In contrast, inhibition of other miRNAs (e.g., miR-107, miR-30a, miR-29, miR-212-3p, and miR-132-3p) exacerbated Aβ and glutamate toxicities (Fig. 1a), suggesting neuroprotective functions for this group of miRNAs. No correlation was observed between the expression levels of these miRNAs [57, 66] and their neuroprotective activities (Fig. S1e).

**Fig. 2:**
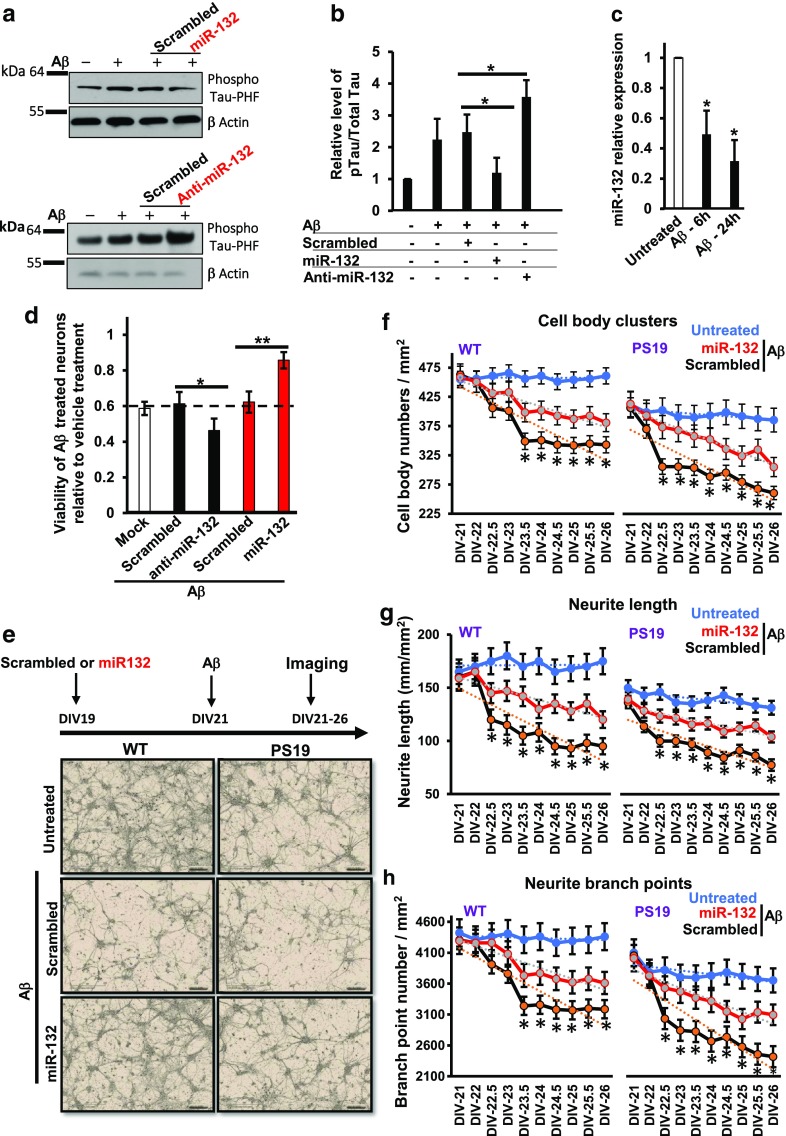
MiR-132 recues morphology and improves health of WT and Tau P301S neurons treated with toxic Aβ species. **a** Western blot analysis of phospho-Tau (PHF1) in mouse primary neurons transfected with either miR-132 mimic, inhibitor, or corresponding “scrambled” control oligonucleotides, and treated with Aβ demonstrates that miR-132 reduces PHF1 levels. **b** Quantification of Western blots analyzing three independent experiments. **c** qRT-PCR analysis demonstrates that Aβ reduces miR-132 expression. **d** Relative viability of primary PS19/P301S neurons transfected with either anti-miR-132 or miR-132 mimic and treated with Aβ. The data were normalized to the viability of untreated cells. **e** Live-cell imaging of WT and PS19 neurons, and representative images shown for DIV26. Quantification of the **f** cell body clusters, **g** neurite length, and **h** number of neurite branch points, for 18 images per condition taken between days 23–26, derived from eight primary cultures. Graphical data are shown as mean ± SEM, *n* = 18 per condition, *P < 0.005, Student’s *t* test

To validate miRNAs modulating neuronal survival, we transfected mouse hippocampal neurons with oligonucleotide mimics of the top 12 hits and then added Aβ or glutamate. As expected, most miRNAs whose inhibitors decreased the viability in the primary screen appeared neuroprotective when their mimics were applied (miR-132-3p, miR-212-3p, miR-129-5p, and miR-29a-5p in Fig. 1b). Conversely, the miRNAs whose inhibitors were neuroprotective exacerbated the toxicity (miR-26b and -34a in Fig. 1b). Similar experiments with both miRNA inhibitors and mimics were also performed on human primary cortical neurons stressed with Aβ (Fig. 1c). Of note, we could not establish a reliable and accurate assay for glutamate toxicity in human primary cells, likely due to the significant protective effect of glial cells present in these cultures. The results in the rodent and human cultures were in good agreement for the 12 miRNAs tested, and in both cases, miR-132(-3p) was identified as the most neuroprotective and miR-26b as the most “neurotoxic” miRNA (Fig. 1b, c). Notably, miR-132 mimic increased the survival of both human and mouse neurons treated with Aβ by ~ 20% (Fig. 1a–c). We also observed that Aβ-induced Tau hyperphosphorylation, which was essential for Aβ neurotoxicity [49], was restrained by miR-132 overexpression and exacerbated by its inhibition (Fig. 2a, b). At the same time, exposure to Aβ reduced neuronal miR-132 expression already at 6 h post-treatment (Fig. 2c).

### Overexpression of miR-132 preserves cell body clusters and neurite integrity in WT and PS19 neurons treated with ½ *t*_max_ Aβ

To investigate the protective effects of miR-132 overexpression in vitro and in vivo, we used a PS19 tau transgenic mouse line which expresses human 1N4R Tau bearing the P301S mutation associated with FTD [68]. PS19 primary neurons were transfected with either anti-miR-132, miR-132 mimic, or control oligonucleotides, and then treated with Aβ. As with WT neurons, in PS19 primary neurons, the miR-132 mimic protected against Aβ, while the anti-miR-132 exacerbated sensitivity to Aβ (Fig. 2d). To investigate the effects of miR-132 overexpression on neuronal morphology in normal versus Aβ-stressed cultures, we imaged live cells over a 5-day interval (Fig. 2e). Of note, naïve PS19 cultures appeared less healthy than WT cultures, exhibited fewer and more clustered cell bodies, and had shorter and less branched neurites. In both WT and PS19 neurons stressed with ½*t*_max_ Aβ, miR-132 mimic increased the number of healthy cell bodies, neurite length, and branch points versus neurons transfected with scrambled oligonucleotides (Fig. 2f–h). These data demonstrate that, under stress or toxic conditions, miR-132 rescues neuritic loss and helps to maintain neuronal integrity in both WT and mutant Tau neurons.

### miR-132 reduces the levels of total and post-translationally modified forms of Tau, its cleavage, and release in PS19 neurons

**Fig. 3:**
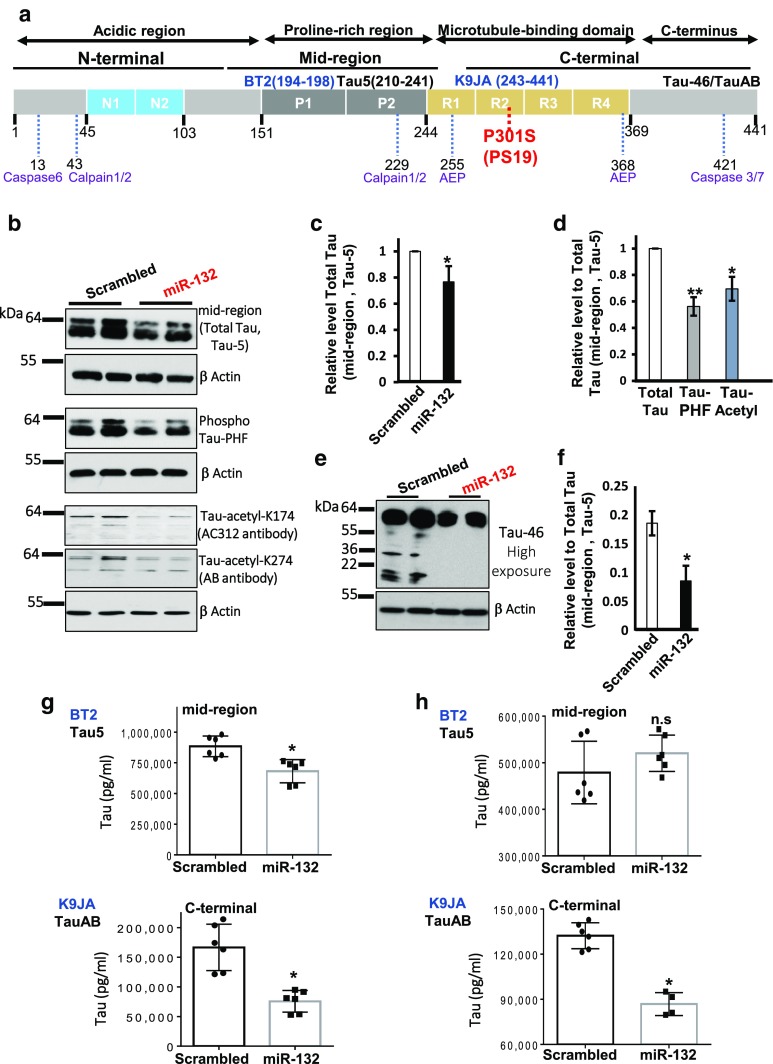
MiR-132 reduces the levels of total Tau, its phosphorylated, and acetylated forms, Tau cleavage and release of a C-terminal fragment. **a** Schematic presentation of Tau protein domains, positions of the epitopes recognized by the antibodies utilized in this study, and proteolytic sites of major proteases. The longest human CNS isoform, Tau 441, is depicted. **b**–**f** Primary PS19 cortical neurons were transfected with either miR-132 mimics or scramble control oligonucleotides, and protein lysates collected 48-h post-transfection and analyzed. β-actin served as a loading control for all Western blots. **b** Western blots analysis demonstrate the effects of miR-132 mimic on the levels of total Tau, and its phosphorylated and acetylated forms. Primary antibodies used are indicated in the parentheses. **c** Quantification of the total Tau in neurons transfected with miR-132, plotted relative to the levels in neurons transfected with scrambled oligonucleotides. **d** Quantification of phosphorylated and acetylated Tau in neurons transfected with miR-132, plotted relative to the levels of total Tau (set as “1”), *P < 0.05, and **P < 0.005, *n* = 12 cultures per condition, *t* test. **e** Western blots analysis demonstrates that miR-132 reduces Tau fragmentation. **f** Quantification of three Western blot experiments similar to that shown in **e**. **g** Quantification of mid-region (left panel) and C-terminal (right panel) containing forms of Tau in neuronal lysates, measured by the BT2-Tau5 and K9JA-TauAB ELISAs (*n* = 6 for each group). **h** Quantification of mid-region (left panel) and C-terminal (right panel) containing forms of Tau in neuronal conditioned medium, measured by ELISA assays (*n* = 6 for each group). *P < 0.005, Student’s *t* test, and *n* = 3. *n.s.* not significant. All graphical data are shown as mean ± SEM (see also Figure S2)

miR-132 downregulation is significantly associated with human Tau pathology [52], and its genetic deficiency increases Tau expression, phosphorylation, and aggregation in 3 × Tg AD transgenic mice [56]. We, therefore, investigated the effects of miR-132 mimic on Tau metabolism, which, in disease, is dysregulated at multiple levels. Tau hyperphosphorylation is a hallmark of most tauopathies [27], but several other post-translational modifications are also common; for instance, acetylation at Lys174 (K174) which hinders the interaction between Tau and microtubules and is thought to foster Tau accumulation and aggregation [9, 39]. In addition, Tau can undergo proteolytic cleavages which generate fragments (Fig. 3a) [18, 51], some of which are prone to aggregation and suggested to be toxic [44, 69].

Western blot analysis revealed that PS19 primary neurons transfected with miR-132 exhibited slightly reduced levels of total Tau and substantial reduction of Tau phosphorylated at Ser396 and Ser404 (PHF1 epitope) and acetylated at K174 and K274 (Fig. 3b–d). Quantification of three independent experiments indicated that the reduction of post-translationally modified Tau isoforms was more pronounced than that of total Tau (Fig. 3d). These data indicate that the observed decrease in the levels of phosphorylated and acetylated Tau was not merely a consequence of reduced total Tau. Additional analysis of major Tau fragments in PS19 neurons using an antibody against the C-terminal region (Tau46) revealed that miR-132 also reduced the levels of ~ 36 and ~ 17 kDa Tau fragments (Fig. 3e, f), the latter being previously characterized as a potentially neurotoxic fragment(s) produced by Calpain 2 and Caspase 3 proteolytic activities, significant amounts of which were found in the brains of patients with tauopathies [13, 14, 50, 51]. Two distinct sandwich ELISA assays, one based on Tau mid-region detection, reflective of total Tau, and the other based on the detection of C-terminal fragments (capture and detection antibodies are illustrated in Fig. 3a), confirmed that miR-132 produces a small (~ 20%) but a significant reduction of the levels of mid-region-containing Tau, and stronger reduction in the levels of C-terminal-containing Tau (Fig. 3g).

It is now widely appreciated that Tau exists both inside and outside of neurons [5, 28, 47]. Under normal circumstances, the majority of extracellular Tau is C-terminal truncated [28, 38, 61], but it has been speculated that, in disease, the C-terminally truncated forms of Tau are released and may contribute to the seeding and spreading of tau aggregates [16]. Since miR-132 diminishes C-terminal Tau fragments inside primary neurons, we next asked whether it may also affect the release of Tau. Notably, although the concentrations of extracellular mid-region-containing tau were unaffected by miR-132 (Fig. 3h, top), the levels of extracellular C-terminal-containing secreted fragments were strongly reduced (Fig. 3h, bottom). Collectively, these results indicate that Tau homeostasis is regulated by miR-132 at several levels, including the regulation of its post-translational modifications, cleavage, and release from neurons.

### miR-132 directly targets the Tau modifiers Rbfox1, GSK3β, EP300, and Calpain 2

**Fig. 4:**
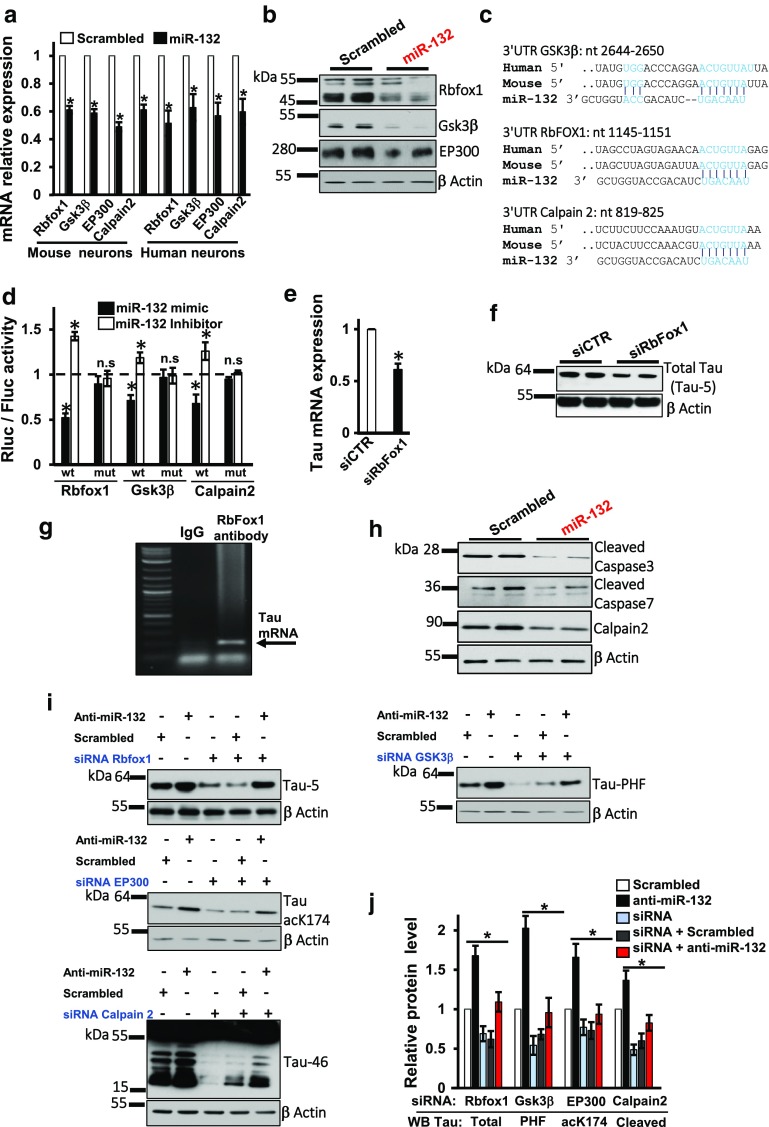
MiR-132 directly targets Tau modifiers Rbfox1, GSK3β, EP300, and Calpain 2. **a** qRT-PCR analysis demonstrates the effects of miR-132 mimic on the expression levels of GSK3β, EP300, Rbfox1, and Calpain 2 mRNAs in WT mouse and human neurons (*P < 0.005, *n* = 6, Student’s *t* test). **b** Effects of miR-132 mimic on protein levels of GSK3β, Rbfox1 and EP300. **c** Predicted miR-132-binding sites within the *GSK3β*, *Calpain 2*, and *RbFOX1* 3′ UTRs. **d** Luciferase reporter assays demonstrate that *RbFOX1*, *Gsk3β,* and *Calpain 2* are direct miR-132 targets, modulated inversely by the miR-132 mimic and inhibitor. Relative activity of luciferase constructs bearing either wild-type (wt) or mutant (mut) *RbFOX1*, *GSK3β*, or *Calpain 2* 3′ UTRs in neural cells co-transfected with miR-132 mimic or inhibitor is presented as renilla/firefly luminescence units (RLU). The data were normalized to the effects of the corresponding control oligonucleotides (set as “1”). *P < 0.005 and *n* = 3; *n.s.* not significant. Graphical data are shown as mean ± SEM. **e** qRT-PCR and **f** Western blot analyses demonstrate that Rbfox1 silencing reduces the levels of total Tau mRNA and protein, respectively, in WT neurons. *P < 0.01, *n* = 3, Student *t* test. **g** iCLIP demonstrates that total Tau mRNA is co-immunoprecipitated with Rbfox1 protein from WT neurons. The immunoprecipitated RNA was amplified by RT-PCR and visualized by gel electrophoresis. **h** Western blot analysis demonstrates that miR-132 reduces the levels of neuronal calpain 2 and cleaved caspase-3 and caspase-7. **i** Western blot analyses and **j** quantification of three experiments demonstrate that the effects of miR-132 inhibition on Tau and its modifications can be rescued by siRNAs to RbFox1, GSK3β, EP300, or Calpain 2

To discover among putative miR-132 targets those that regulate Tau metabolism, we systematically applied several target prediction algorithms. Tau mRNA itself has been proposed as a direct target of miR-132 [56]; however, the previous work failed to confirm this in human neural cells [64]. Here, we identified several molecules that directly link miR-132 to Tau. mRNA for Glycogen Synthase Kinase-3 β (GSK3β) that contributes to pathologic Tau hyperphosphorylation in AD [15, 58] contains two conserved putative miR-132-binding sites within its 3′UTR. Transfections of primary neurons with miR-132 mimic reduced GSK3β amounts at both the mRNA and protein levels (Fig. 4a, b). Furthermore, a luciferase reporter containing the wild-type GSK3β 3′ UTR was repressed by the miR-132 mimic and upregulated by the miR-132 inhibitor. These effects on the reporter were abrogated by a mutation in one of the predicted binding sites at the position 2644–2650 (Fig. 4c, d). These data provide evidence that miR-132 directly binds to the GSK3β mRNA and downregulates its expression, which results in reduced GSK3β activity and GSK3β-mediated phospho-Tau (Fig. S3).

Acetyltransferase EP300 is the major acetylase of Tau at K174 implicated in its aggregation and neurodegeneration in AD [39]. It has been previously reported as one of the miR-132 targets contributing to its pro-survival/anti-apoptotic function [67]. Indeed, miR-132 mimics reduced expression of EP300, at both mRNA and protein levels in primary neurons (Fig. 4a, b). These data suggest that the observed miR-132 effects on Tau acetylation (see Fig. 3b, d) could be directly mediated by EP300.

Rbfox1, also known as Ataxin-2-binding protein 1, is an RNA-binding protein (RBP) that plays a pivotal role in alternative splicing, mRNA stability, and translation in the brain [1, 32]. Rbfox1 was predicted as another highly scored miR-132 target; indeed, miR-132 repressed both Rbfox1 mRNA and protein (Fig. 4a, b). Furthermore, the direct binding and reciprocal regulation of Rbfox1 by miR-132 mimic and inhibitor were validated using the luciferase reporters bearing the WT and mutant Rbfox1 3′UTR, as described above for the GSK3β (Fig. 4c, d). We hypothesized that Rbfox1 may regulate Tau mRNA splicing and/or stability. Indeed, silencing of Rbfox1 by RNAi reduced total mRNA and protein levels of Tau in primary neurons (Fig. 4e, f). Using iCLIP approach (Fig. S4a), we determined that Rbfox1 directly binds to Tau mRNA (Fig. 4g), preferentially via the GCAUG motif site found in its coding region (Fig. S4b, c). Therefore, while the exact molecular mechanism remains to be established, Rbfox1 appears as a novel RBP that promotes Tau expression. All together, these data strongly suggest that miR-132 directly regulates Rbfox1 and thereby reduces Tau mRNA stability and/or translation.

Finally, we observed that several proteases implicated in Tau cleavage, including Caspases 3 and 7, and Calpain 2, are regulated by miR-132 (Fig. 4h). One of them, Calpain 2, has been predicted as miR-132 target (Fig. 4c). qRT-PCR analysis and luciferase reporter assays confirmed, respectively, that miR-132 reduced Calpain 2 mRNA expression (Fig. 4a), and this effect was mediated by the direct miR-132 binding to the Calpain 2 3′UTR (Fig. 4d). Therefore, miR-132 regulates Calpain 2 expression and may, thereby, regulate Tau cleavage. Additional rescue experiments on neurons co-transfected with anti-miR-132 and siRNAs cognate to either Rbfox1, GSK3β, EP300, or Calpain 2 demonstrated that these targets, indeed, mediated miR-132 control of tau levels, modifications, and cleavage, respectively (Fig. 4i, j). Overall, these data indicate that miR-132 regulates Tau modifiers, including GSK3β, EP300, Rbfox1, and Calpain 2 that collectively contribute to Tau homeostasis in neurons. In addition, Caspases 3/7-mediated cleavage of Tau may also be regulated by miR-132 via PTEN/AKT/FOXO3A signaling [67].

**Fig. 5:**
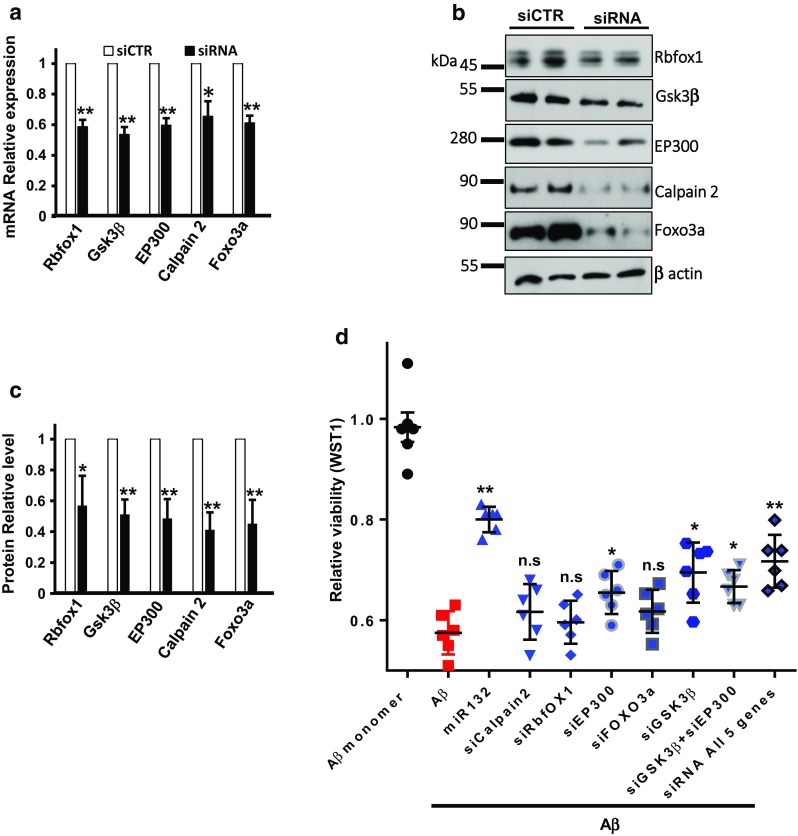
MiR-132 provides stronger neuroprotection than downregulation of its individual targets. **a** mRNA and **b** protein levels of Rbfox1, GSK3β, EP300, Calpain2, and Foxo3a in neurons transfected with cognate siRNAs. **c** Quantification of three Western blot experiments similar to that shown in **b**. **d** Relative viability of primary PS19 neurons transfected with miR-132 mimic or individual siRNAs to Foxo3a, EP300, Calpain2, Rbfox1, Gsk3β, or siRNAs to all five genes, and exposed to toxic Aβ. *P < 0.05, **P < 0.005, *n* = 6. Graphical data are shown as mean ± SEM

To further investigate whether any of the direct miR-132 targets play a dominant role in neuroprotection against Aβ, we compared the effects of miR-132 mimic to that of individual siRNAs cognate to their targets (GSK3β, EP300, Calpain 2, Rbfox1, and previously validated Foxo3a). To mimic target repression provided by miR-132, siRNA concentrations and transfection conditions were optimized to ensure 100% transfection efficiency in P301S neurons with similar level of downregulation for target mRNAs and proteins (Fig. 5a–c). Downregulation of EP300 and GSK3β resulted in a slightly but significantly improved viability of neurons treated with ½*t*_max_ Aβ, while the individual downregulation of Calpain 2, Rbfox1, and Foxo3a was insufficient to reduce the Aβ toxicity. Transfections of the five siRNAs together enhanced the neuroprotection relative to the effects of individual siRNAs, but still did not reach the level of protection provided by miR-132 (Fig. 5c). These results indicate that the neuroprotective properties of miR-132 are mediated by multiple target genes, rather than a single key target, and, perhaps, additional genes beyond the five studied here.

### Overexpression of miR-132 in PS19 mice reduces caspase-3 activation, Tau hyperphosphorylation, and neuron loss

**Fig. 6:**
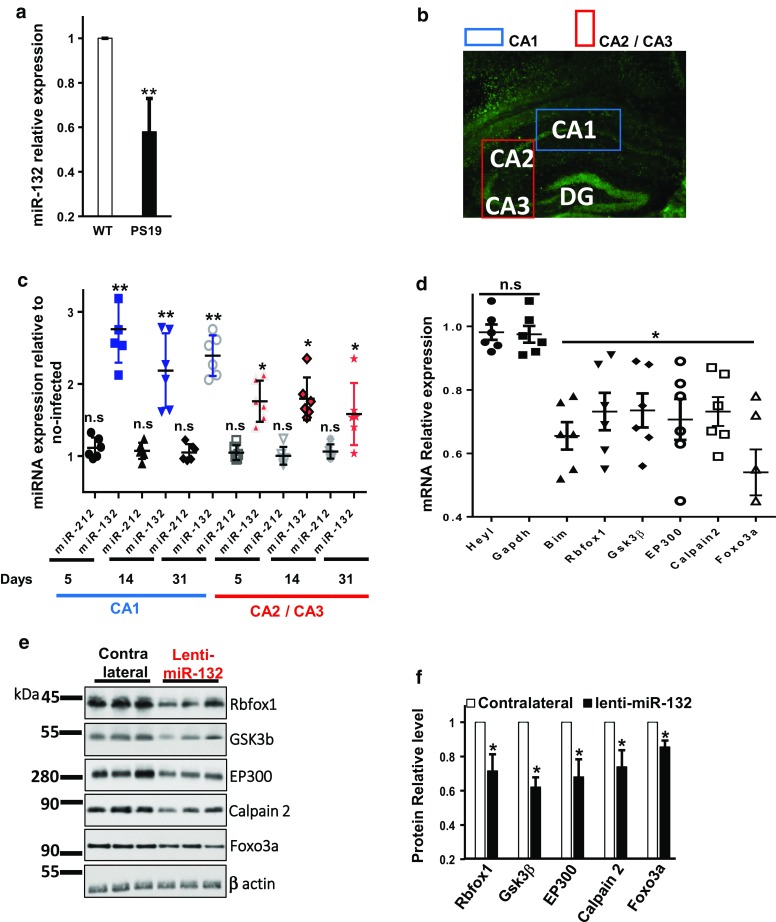
Stable supplementation of miR-132 and downregulation of its targets in the murine hippocampus. **a** qRT-PCR analysis demonstrates reduced expression of miR-132 in the PS19 CA1 versus its expression in the CA1 area of 6-month-old WT mice. **b** NeuN IHC of the mouse hippocampal CA1 area, injected with the miR-132-expressing lentivirus (6 × 10^6^ TU/ml**)**, and the adjacent CA2/CA3 regions. The boxes depict the areas microdissected for the analysis. **c** qRT-PCR analysis demonstrates elevation of miR-132 (but not genomically co-encoded miR-212) in the CA1 and CA2/CA3 regions at days 5, 14, and 31 post-injection of the LV-miR132, relative to the EV (*n* = 6). **d** qRT-PCR and **e** Western blot analyses shows the reduced expression of miR-132 targets Rbfox1, Gsk3β, EP300, Calpain2, Foxo3a, and downstream Bim but not of control genes, in the CA1, at day 31 post-injections. **f** Quantification of two Western blot experiments similar to that shown in **e**. *P < 0.05, **P < 0.005, *n* = 6. Graphical data are shown as mean ± SEM (see also Figure S4)

Neurons in AD exhibit a ~ two-to-threefold downregulation of miR-132 levels [31, 67] and similar downregulation was observed in the hippocampus of the 6-month-old PS19 mice (Fig. 6a). To investigate the neuroprotective properties of miR-132 in vivo, we produced a lentivirus expressing the mature miR-132 from the Synapsin promoter (Lenti-miR132), and a control virus lacking the miR-132 gene (Lenti-Empty) (Fig. S5). The conditions for durable and spatially defined miR-132 overexpression were optimized for the titrated Lenti-miR132 injected stereotactically into the hippocampal CA1 area of 6-month-old wild-type mice (Fig. 6b). The levels of miR-132 overexpression were assessed in the CA1 and the adjacent CA2/CA3 at days 5, 14, and 31 post-injection (Fig. 6c). To reduce non-specific effects and avoid saturation of the system, virus titers of 10^6^ transducing units (TU)/ml provided stable ~ 2.5-to-3-fold miR-132 overexpression in CA1 and ~ 1.5-to-2-fold in CA2/CA3 neurons for at least 1 month were selected for the subsequent experiments (Fig. 6c). This increase in miR-132, but not its paralog miR-212, led to a corresponding downregulation of validated miR-132 targets, at mRNA and protein level, including pro-apoptotic FOXO3a, EP300, and downstream effector Bim, and newly established targets Rbfox1, GSK3β, and Calpain 2 (Fig. 6d–f). Therefore, this lentivirus system provides sustained functional miR-132 overexpression in mouse hippocampus.

**Fig. 7:**
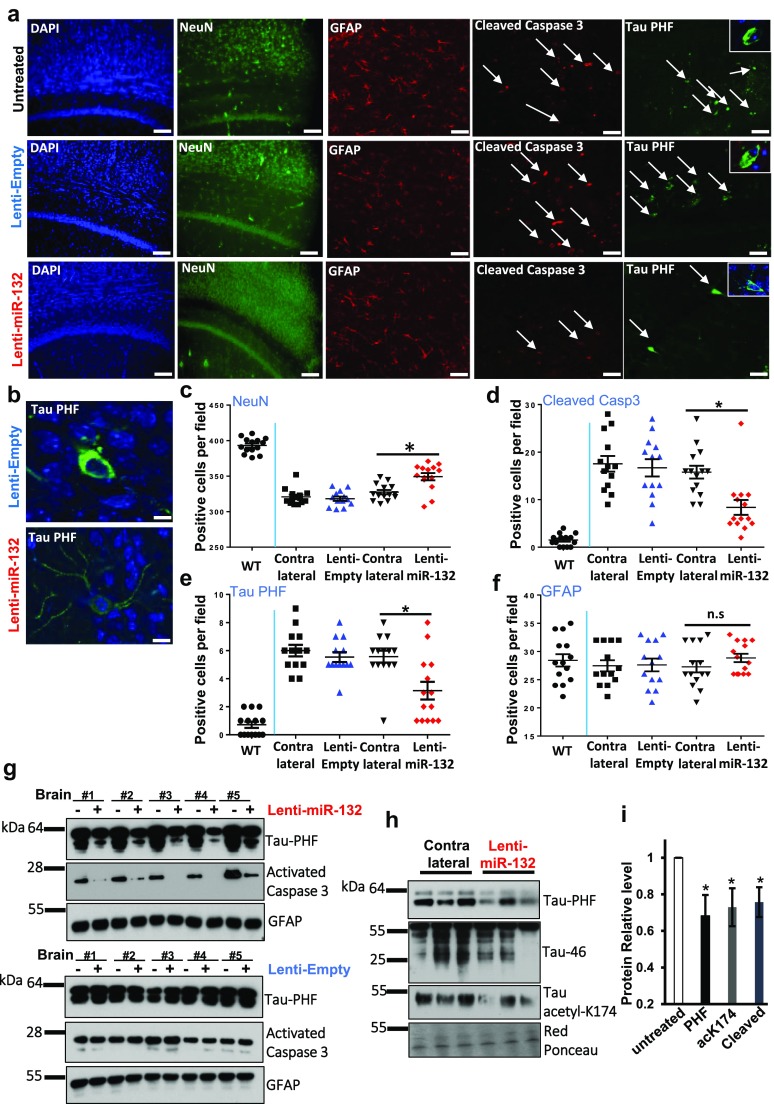
miR-132 supplementation reduces Tau pathology and neuronal loss in PS19 mice. **a** Representative IHC images of CA1 and adjacent cortical layers stained for NeuN, cleaved Caspase-3, PHF-Tau, glial fibrillary acidic protein (GFAP), and DAPI staining. The cells positive for the activated caspase-3 and PHF-Tau are marked by arrows. Scale bar = 100 µm. **b** Tangle-like PHF-positive inclusions (in green) observed in the cell bodies of untreated/EV-treated neurons, and the less intense and more evenly distributed PHF staining in the soma and neurites of apparently intact neurons from miR-132-treated mice. Scale bar = 100 µm. **c**–**f** Image J quantification of the marker-positive cells per field in the CA1 area. For all images and quantifications, *n* = 14 mice per condition, 15 sections per brain. All graphical data are shown as mean ± SEM, Student’s *t* test, *P < 0.005. **g** Western blot analyses of hippocampal specimens from the brains injected with LV-miR132 or EV. “+” corresponds to the injected hemisphere and “−” to the contralateral part. **h** Western blot analyses and **i** quantification of Tau-PHF, cleaved, and acetylated Tau in the sarkosyl-insoluble brain fractions

We then investigated the neuroprotective effects of Lenti-miR132 in PS19 mice, which develop NFT-like inclusions in the brain and spinal cord starting at around 6 months of age and evince neuronal loss and brain atrophy by 8 months [36]. In the first set of experiments, the viruses were stereotactically injected into the CA1 hippocampal area twice, first at 7.5 months, and then at 9 months of age. Three experimental groups that included untreated animals, and mice injected to the right hippocampus with either Lenti-empty or Lenti-miR132, were analyzed in parallel (15 mice per group). The animals were sacrificed at 10.5 months, and the brains analyzed by IHC and quantitative image analysis for key markers, including the PHF-Tau, NeuN, activated Caspase-3, and GFAP, and further by Western blotting (Fig. 7a–g). There were ~ 19% fewer NeuN-positive neurons in the hippocampal CA1 areas and adjacent cortical layers of PS19 mice than in the WT brains (Fig. 7a, c). PHF-Tau (a marker of pathology) as well as cleaved caspase-3 (a marker of apoptosis) were evident in all PS19 brain sections, but absent in WT brains (Fig. 7a, d, e and Fig. S6). Lenti-miR132 significantly reduced the numbers of cells positive for PHF-Tau and cleaved caspase-3 in comparison to the contralateral hemispheres, and to the brains injected with the empty virus, or left untreated (Fig. 7a, d, e, g). The levels of sarkosyl-insoluble PHF-Tau, cleaved, and acetylated Tau were also reduced by miR-132 (Fig. 7h, i). Correspondingly, image quantification indicated that Lenti-miR132 increased the number of NeuN-positive hippocampal neurons in PS19 mice (Fig. 7c), but GFAP staining was not different between the PS19 groups (Fig. 7a, f, g). Consistent with the reduced Tau pathology and neuronal apoptosis, miR-132 increased hippocampal volume relative to the Lenti-Empty group (Fig. S7). These results demonstrate a significant in vivo neuroprotection provided by miR-132 in PS19 mice in the progressive stage of neurodegeneration.

In an additional set of experiments, PS19 mice were stereotactically injected with Lenti-miR132 a total of three times (i.e., at 3, 4.5, and 6 months, 7 mice per group) before onset of pathology, and analyzed at a time point when pathology is obvious in untreated PS19 mice (i.e., 10 months). In accord with our “treatment” study, overexpression of miR-132 prevented neuronal loss and accumulation of PHF-Tau when administered prior to the emergence of tau pathology (Fig. S8). Thus, dependent on the time of administration, neuronal miR-132 can prevent or halt Tau pathology and neurodegeneration.

### Overexpression of miR-132 enhances hippocampal LTP in WT mice and restores it in PS19 mice

**Fig. 8:**
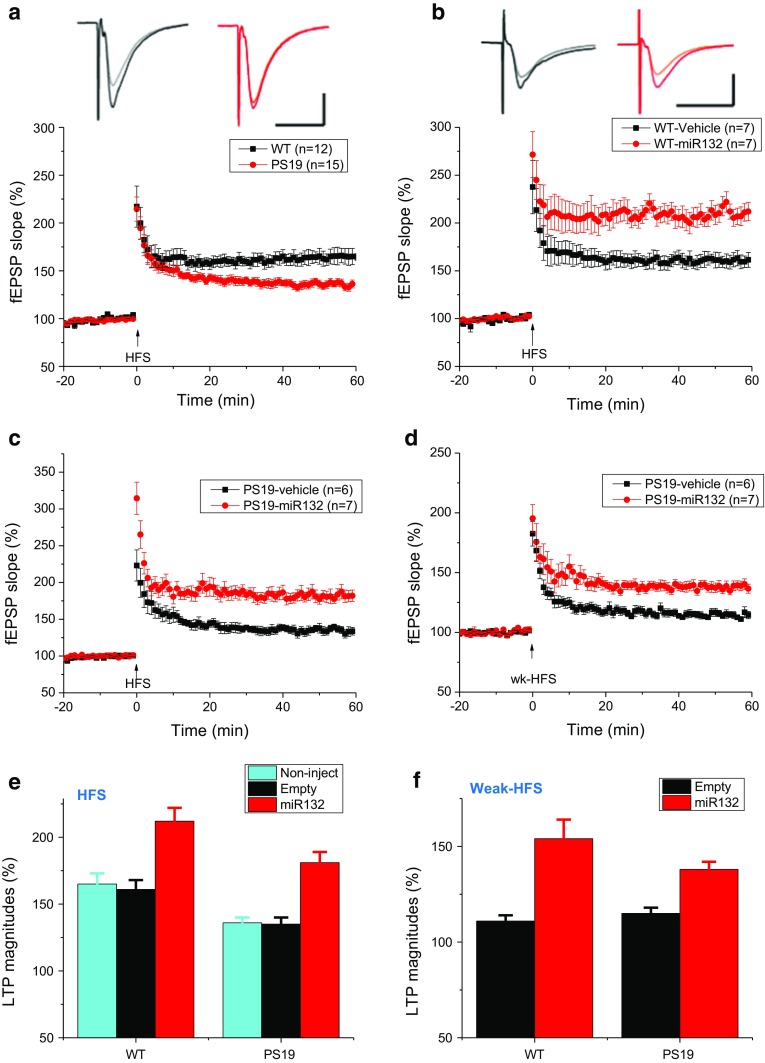
Hippocampal recording demonstrates that miR-132 supplementation rescues LTP impairment in the PS19 mice. **a** Percentage of potentiation of field EPSPs recorded before and after tetanic stimulation of Schaffer’s collaterals in brain slices of PS19 transgenic mice (red) and littermate wild-type mice (black). Each data point shown is the mean ± SEM of results from eight individual mice of each genotype. **b** Hippocampal miR-132 injection significantly increased LTP induced by high-frequency stimulation (HFS, arrow) in the CA1 region of hippocampal slices (red circles, *n* = 7 slices/from 5 mice) vs. those of mice in vehicle (black squares, *n* = 7/4). **c** Reversing effect of miR132 on LTP induced by high-frequency stimulation (HFS, 100 Hz last 1 s, two trains separated by 20 s) in PS 19 mice injected with either empty lentiviruses (black squares, *n* = 6/4) or LV-miR132 (red circles, *n* = 7/4). **d** miR132 injection enable a weak HFS (100 Hz last 1 s) induced LTP in PS19 mice injected with LV-miR132 (red circles, *n* = 7/5) not the empty lentiviruses (black squares, *n* = 6/4). Bar diagram summarizing the LTP experiments with WT and PS19 mice induced by standard HFS (**e**) or weak HFS (**f**). Inset traces are typical field excitatory postsynaptic potentials (fEPSPs) recorded before (gray or orange) and after (black or red) HFS for each condition. Horizontal calibration bars: 10 ms; vertical bars: 0.5 mV

To further investigate functional effects of miR-132 supplementation in the brains of PS19 mice, we measured hippocampal long-term potentiation (LTP), an electrophysiology correlate of learning and memory. Standard high-frequency stimulation (HFS) was applied to the CA1 hippocampal region of WT and PS19 mice, and average percent changes in fEPSP slopes relative to baseline stimulation were plotted over time post-HFS (*n* = 7–15) (Fig. 8a, b). As expected for mice exhibiting neuronal loss [68] LTP was consistently lower in brain slices from 7.5-month PS19 mice than in age-matched WT mice (136 ± 4%, *n *= 15 vs. 165 ± 8%, *n *= 12, *P* < 0.001; Fig. 8a). In WT mice, LTP was enhanced in animals injected with LV-miR132 more than in those injected with empty LV (212 ± 10%, *n *= 7 vs. 161 ± 7%, *n *= 7, *P* < 0.001; Fig. 8b). In PS19 mice, LV-miR132 treatment dramatically increased LTP to levels greater or comparable to that of untreated WT mice (181 ± 8%, *n *= 7 vs. 135 ± 5%, *n *= 6, *P* < 0.001; Fig. 8c–e). LV-miR132 treatment also potentiated the effects of weak HFS that was insufficient to induce significant LTP in hippocampal slices (115 ± 3 vs. 138 ± 4%, *P* < 0.001; Fig. 8f).

## Discussion

Several lines of evidence implicate reduced miR-132 activity in AD and related neurodegenerative conditions. First, from many independent attempts to define miRNAs linked to AD pathology, miR-132 has emerged as the top molecule significantly associated with both plaques and tangles in a variety of disease affected brain areas [21, 22, 24, 45, 53, 64, 67]. miR-132 is downregulated starting at Braak III stage, before neuron loss, and miR-132 reduction is evident in phospho-tau-positive neurons [31, 53]. Furthermore, miR-132 downregulation has been described in other neurodegenerative disorders linked to aggregation and accumulation of misfolded protein Tau, including frontotemporal lobar degeneration and progressive supranuclear palsy [6, 21, 55]. Although miR-132 downregulation in the latter classes of tauopathies still requires validation in larger brain cohorts, the data suggest a possible common mechanism underlying miR-132 dysregulation in both AD and primary tauopathies. Second, miR-132 knockout impairs memory formation and retention in adult mice, induces Tau aggregation, and aggravates both tau and amyloid pathologies in transgenic mouse models [22, 31, 53]. Third, our high-content screen for miRNA modulators of neuroprotection against Aβ and glutamate excitotoxicity performed in this study, identified miR-132 as the top hit (Fig. 1). Finally, miR-132 has important regulatory functions in neuron development, synaptic plasticity, and survival. Of note, additional neuroprotective (e.g., miR-29 and miR-129) and “neurotoxic” (e.g., miR-26b and miR-34a) miRNAs identified in our screen have been previously implicated in the regulation of critical genes and pathways in AD [20, 41, 45]; it will be important to investigate these hits in future studies.

Several targets and signaling pathways may underlie miR-132 neuroprotective functions. Some of them, such as p250GAP, RASA1, and MeCP2, mediate the role of miR-132 in neurite extension, arborization, and synaptogenesis [63]. Other targets, such as PTEN, p300, and FOXO3a, counteract AKT pro-survival signaling; their derepression observed in AD neurons and probably caused by miR-132 downregulation may induce expression of the key apoptotic effectors Bim and Puma, leading to activation of caspases and apoptotic signaling [67], and also promoting Tau cleavage. Furthermore, recent reports suggest certain direct miR-132 targets implicated in Aβ and Tau metabolism, including the Tau mRNA itself [56]. However, miR-132 does not appear to regulate Tau in human neurons directly [64]. Tau homeostasis is tightly controlled at multiple levels, and we report here that miR-132 regulates key factors affecting tau production, post-translational modifications, and proteolysis. Specifically, miR-132 regulates tau phosphorylation (via direct targeting of GSK3β), acetylation (via a EP300), and cleavage (through calpain 2 and caspases-3/7), and it also reduces Tau mRNA via the direct targeting of the RNA-binding protein, Rbfox1. Tau hyperphosphorylation at PHF1 epitope, largely mediated by GSK3β, affects microtubule dynamics and NFT accumulation, which is considered a hallmark cytopathology in AD and other tauopathies [23]. Since we validated both major tau kinase GSK3β and acetylase EP300 as the direct miR-132 targets (Fig. 4, and [67]), and additional tau kinase CDK5 is also indirectly repressed by miR-132 via NOS1 signaling [64], thus, miR-132 emerges as the major regulator of the post-translational modifications of Tau.

Our work also demonstrates that miR-132 regulates Tau cleavage and implicates a newly validated target calpain 2, as well as caspases 3 and 7, in this event. Tau is cleaved by multiple proteolytic enzymes which facilitate its degradation and clearance. However, if allowed to accumulate, some of these fragments become aggregated and/or hyperphosphorylated and neurotoxic [18, 51]. For instance, Tau cleavage by calpain 2 produces a 17 kDa neurotoxic fragment, and significant amounts of these fragments are found in the brains of patients with tauopathy [14, 50]. Mutations in calpain in transgenic flies were shown to prevent Tau toxicity [50]. In addition to the proteolysis of Tau, Calpain also cleaves p35, the principal activator of Cdk5, into p25, which results in the hyperactivation of both Cdk5 and GSK3β, and thereby induces tau hyperphosphorylation [8, 30]. Here, we demonstrate that miR-132 not only regulates Calpain 2 directly, but its levels also inversely correlate with the levels of Calpain 2 mRNA in hundreds of AD brains, suggesting that miR-132 is a primary regulator of Calpain 2 expression in the brain, responsible for its upregulation in AD. In addition, caspase 3/7 activity, modulated by miR-132 indirectly, likely through PTEN/FOXO3/Bim signaling [67], may also contribute to Tau cleavage and the observed release of tau fragments from neurons (Fig. 3d, e).

We also validated Rbfox1, an RNA-binding protein highly expressed in neuronal tissues, as another direct miR-132 target. By binding to the GCAUG element, Rbfox1 plays a pivotal role in alternative splicing, mRNA stability, and translation [1, 32]. The Rbfox1 knockout mice have a significant increase in neuronal excitability in the dentate gyrus [19], and a recent study identified a link between Rbfox1 protein and AD [1]. We demonstrate that Rbfox1 binds to and stabilizes neuronal Tau mRNA. Altogether, considering the additional miR-132 target PTBP2 previously implicated in Tau mRNA splicing [55], these data position miR-132 as the principal regulator of various aspects of Tau homeostasis provide a mechanistic link between the miR-132 downregulation and Tau pathology observed in disease.

Overall, our work supports miR-132 as the master regulator of neuronal health. In addition to its distinct functions in synaptogenesis, neuronal activity, plasticity, memory, and neuronal viability [37, 63], miR-132 regulates Tau metabolism, and its downregulation in AD and other neurodegenerative diseases likely promotes pathogenesis by perturbing multiple signaling pathways. Interestingly, an initial increase in miR-132 levels during early AD Braak stages I–II in the human prefrontal cortex has been described, which contrasts with the decrease seen at more advanced stages of the disease [31]. A similar bi-phasic miR-132 expression pattern has been reported in prion disease [34], suggesting that miR-132 is part of an initial neuroprotective response. Subsequent downregulation, however, can aggravate the effects of Aβ and Tau toxicities [22]. Of note, miR-132 is regulated by the activity-dependent cAMP-response element-binding (CREB) transcription factor, and its expression pattern in the AD brain mimics that of the brain-derived neurotrophic factor [29]. In addition, alterations in DNA methylation that affect gene expression and perhaps the onset of AD [11] may play a role in miR-132 downregulation in neurons, as demonstrated for some cancer cells [17].

Collectively, these data suggest that miR-132 replacement in tauopathies, such as AD, may provide a much-desired neuroprotective effect. Lowering Tau alone with antisense oligonucleotides (ASO) has recently been shown as therapeutically beneficial for tauopathies [12]. Here, we provide a proof-of-principle for miR-132 replacement as a novel neuroprotective strategy to reduce Tau pathology and simultaneously promote nerve growth and regeneration, enhance neuronal survival, and, consequently, improve cognition. miR-132 supplementation protects against strong toxic stimuli even in highly vulnerable and damaged Tau-mutant neurons, in vitro and in vivo. It had preventive effects in young presymptomatic PS19 mice and reduced neuronal loss and Tau pathology even when pathology was already established. The PS19 model exhibits broad brain and spinal cord pathology resulting in severe cognitive, motor, and visual impairments [59, 68]. Since our proof-of-concept study relies on the lentivirus-mediated local unilateral miR-132 supplementation to the CA1 region, it did not allow examination of the effects on global readouts such as neurologic and behavioral phenotypes. To overcome this limitation, broader distribution of miR-132 mimics in the CNS will be required.

Notably, small molecules and other types of inhibitors of major miR-132 targets have entered clinical trials. These include inhibitors of GSK3β, EP300, Calpains, as well as Tau-targeting antibodies and ASO drugs [24, 48, 60, 70]. Remarkably, miR-132 is the natural inhibitor of each of these factors. Therefore, its replacement can provide a multi-hit approach and ensure the benefits of combination therapies. MiR-132 replacement strategies for tauopathies will largely rely on the development of miRNA-mimicking oligonucleotides and technologies for their delivery to the brain and leverage recent advances in the field of oligotherapeutics. Notably, the first “breakthrough” oligonucleotide-based drug for a neurologic disease has recently gained fast FDA approval [43], and many more are at different stages of clinical development for a wide spectrum of neurodegenerative disorders. AD and other tauopathies have so far proven refractory to small molecules and biological drugs, and miRNA mimics emerge as a new and promising class of therapeutics. Our work validates miR-132 as a first-line candidate for development of such neurotherapies.

## Electronic supplementary material

Below is the link to the electronic supplementary material.

Supplementary material 1 (PDF 1251 kb)

## References

[1] Alkallas R, Fish L, Goodarzi H, Najafabadi HS (2017). Inference of RNA decay rate from transcriptional profiling highlights the regulatory programs of Alzheimer’s disease. Nat Commun.

[2] Bak M, Silahtaroglu A, Møller M, Christensen M, Rath MF, Skryabin B (2008). MicroRNA expression in the adult mouse central nervous system. RNA.

[3] Bloom GS (2014). Amyloid-β and tau: the trigger and bullet in Alzheimer disease pathogenesis. JAMA Neurol.

[4] Cantlon A, Frigerio CS, Freir DB, Boland B, Jin M, Walsh DM (2015). The familial British dementia mutation promotes formation of neurotoxic cystine cross-linked Amyloid Bri (ABri) oligomers. J Biol Chem.

[5] Chai X, Wu S, Murray TK, Kinley R, Cella CV, Sims H (2011). Passive immunization with anti-Tau antibodies in two transgenic models: reduction of Tau pathology and delay of disease progression. J Biol Chem.

[6] Chen-Plotkin AS, Unger TL, Gallagher MD, Bill E, Kwong LK, Volpicelli-Daley L (2012). TMEM106B, the risk gene for frontotemporal dementia, is regulated by the microRNA-132/212 cluster and affects progranulin pathways. J Neurosci Off J Soc Neurosci.

[7] Chou C-H, Shrestha S, Yang C-D, Chang N-W, Lin Y-L, Liao K-W (2018). miRTarBase update 2018: a resource for experimentally validated microRNA–target interactions. Nucleic Acids Res.

[8] Chow H-M, Guo D, Zhou J-C, Zhang G-Y, Li H-F, Herrup K, Zhang J (2014). CDK5 activator protein p25 preferentially binds and activates GSK3β. Proc Natl Acad Sci USA.

[9] Cohen TJ, Guo JL, Hurtado DE, Kwong LK, Mills IP, Trojanowski JQ, Lee VMY (2011). The acetylation of tau inhibits its function and promotes pathological tau aggregation. Nat Commun.

[10] Davis TH, Cuellar TL, Koch SM, Barker AJ, Harfe BD, McManus MT, Ullian EM (2008). Conditional loss of Dicer disrupts cellular and tissue morphogenesis in the cortex and hippocampus. J Neurosci Off J Soc Neurosci.

[11] De Jager PL, Srivastava G, Lunnon K, Burgess J, Schalkwyk LC, Yu L (2014). Alzheimer’s disease: early alterations in brain DNA methylation at ANK1, BIN1, RHBDF2 and other loci. Nat Neurosci.

[12] DeVos SL, Miller RL, Schoch KM, Holmes BB, Kebodeaux CS, Wegener AJ (2017). Tau reduction prevents neuronal loss and reverses pathological tau deposition and seeding in mice with tauopathy. Sci Transl Med.

[13] Fasulo L, Ugolini G, Visintin M, Bradbury A, Brancolini C, Verzillo V (2000). The neuronal microtubule-associated protein tau is a substrate for caspase-3 and an effector of apoptosis. J Neurochem.

[14] Ferreira A, Bigio EH (2011). Calpain-mediated tau cleavage: a mechanism leading to neurodegeneration shared by multiple tauopathies. Mol Med Camb Mass.

[15] Ferreira A, Lu Q, Orecchio L, Kosik KS (1997). Selective phosphorylation of adult tau isoforms in mature hippocampal neurons exposed to fibrillar A beta. Mol Cell Neurosci.

[16] Florenzano F, Veronica C, Ciasca G, Ciotti MT, Pittaluga A, Olivero G (2017). Extracellular truncated tau causes early presynaptic dysfunction associated with Alzheimer’s disease and other tauopathies. Oncotarget.

[17] Formosa A, Lena AM, Markert EK, Cortelli S, Miano R, Mauriello A (2013). DNA methylation silences miR-132 in prostate cancer. Oncogene.

[18] Gamblin TC, Chen F, Zambrano A, Abraha A, Lagalwar S, Guillozet AL (2003). Caspase cleavage of tau: linking amyloid and neurofibrillary tangles in Alzheimer’s disease. Proc Natl Acad Sci USA.

[19] Gehman LT, Stoilov P, Maguire J, Damianov A, Lin C-H, Shiue L (2011). The splicing regulator Rbfox1 (A2BP1) controls neuronal excitation in the mammalian brain. Nat Genet.

[20] Hébert SS, Horré K, Nicolaï L, Bergmans B, Papadopoulou AS, Delacourte A, De Strooper B (2009). MicroRNA regulation of Alzheimer’s Amyloid precursor protein expression. Neurobiol Dis.

[21] Hébert SS, Wang W-X, Zhu Q, Nelson PT (2013). A study of small RNAs from cerebral neocortex of pathology-verified Alzheimer’s disease, dementia with lewy bodies, hippocampal sclerosis, frontotemporal lobar dementia, and non-demented human controls. J Alzheimers Dis JAD.

[22] Hernandez-Rapp J, Rainone S, Goupil C, Dorval V, Smith PY, Saint-Pierre M (2016). microRNA-132/212 deficiency enhances Aβ production and senile plaque deposition in Alzheimer’s disease triple transgenic mice. Sci Rep.

[23] Hooper C, Killick R, Lovestone S (2008). The GSK3 hypothesis of Alzheimer’s disease. J Neurochem.

[24] Hu S, Begum AN, Jones MR, Oh MS, Beech WK, Beech BH (2009). GSK3 inhibitors show benefits in an Alzheimer’s disease (AD) model of neurodegeneration but adverse effects in control animals. Neurobiol Dis.

[25] Huppertz I, Attig J, D’Ambrogio A, Easton LE, Sibley CR, Sugimoto Y (2014). iCLIP: protein–RNA interactions at nucleotide resolution. Methods.

[26] Idda ML, Munk R, Abdelmohsen K, Gorospe M (2018). Noncoding RNAs in Alzheimer’s disease. Wiley Interdiscip Rev RNA.

[27] Iqbal K, Liu F, Gong C-X, Grundke-Iqbal I (2010). Tau in Alzheimer disease and related tauopathies. Curr Alzheimer Res.

[28] Kanmert D, Cantlon A, Muratore CR, Jin M, O’Malley TT, Lee G (2015). C-terminally truncated forms of tau, but not full-length tau or its C-terminal fragments, are released from neurons independently of cell death. J Neurosci Off J Soc Neurosci.

[29] Klein ME, Lioy DT, Ma L, Impey S, Mandel G, Goodman RH (2007). Homeostatic regulation of MeCP2 expression by a CREB-induced microRNA. Nat Neurosci.

[30] Kurbatskaya K, Phillips EC, Croft CL, Dentoni G, Hughes MM, Wade MA (2016). Upregulation of calpain activity precedes tau phosphorylation and loss of synaptic proteins in Alzheimer’s disease brain. Acta Neuropathol Commun.

[31] Lau P, Bossers K, Janky R, Salta E, Frigerio CS, Barbash S (2013). Alteration of the microRNA network during the progression of Alzheimer’s disease. EMBO Mol Med.

[32] Lee J-A, Damianov A, Lin C-H, Fontes M, Parikshak NN, Anderson ES (2016). Cytoplasmic Rbfox1 regulates the expression of synaptic and autism-related genes. Neuron.

[33] Magill ST, Cambronne XA, Luikart BW, Lioy DT, Leighton BH (2010). microRNA-132 regulates dendritic growth and arborization of newborn neurons in the adult hippocampus. Proc Natl Acad Sci USA.

[34] Majer A, Medina SJ, Niu Y, Abrenica B, Manguiat KJ, Frost KL (2012). Early mechanisms of pathobiology are revealed by transcriptional temporal dynamics in hippocampal CA1 neurons of prion infected mice. PLoS Pathog.

[35] Marler KJ, Suetterlin P, Dopplapudi A, Rubikaite A, Adnan J, Maiorano NA (2014). BDNF promotes axon branching of retinal ganglion cells via miRNA-132 and p250GAP. J Neurosci Off J Soc Neurosci.

[36] Maruyama M, Shimada H, Suhara T, Shinotoh H, Ji B, Maeda J (2013). Imaging of tau pathology in a tauopathy mouse model and in Alzheimer patients compared to normal controls. Neuron.

[37] Mellios N, Sugihara H, Castro J, Banerjee A, Le C, Kumar A (2011). miR-132, an experience-dependent microRNA, is essential for visual cortex plasticity. Nat Neurosci.

[38] Meredith JE, Sankaranarayanan S, Guss V, Lanzetti AJ, Berisha F, Neely RJ (2013). Characterization of novel CSF Tau and ptau biomarkers for Alzheimer’s disease. PLoS One.

[39] Min S-W, Chen X, Tracy TE, Li Y, Zhou Y, Wang C (2015). Critical role of acetylation in tau-mediated neurodegeneration and cognitive deficits. Nat Med.

[40] Minogue AM, Stubbs AK, Frigerio CS, Boland B, Fadeeva JV, Tang J (2009). Gamma-secretase processing of APLP1 leads to the production of a p3-like peptide that does not aggregate and is not toxic to neurons. Brain Res.

[41] Modi PK, Jaiswal S, Sharma P (2016). Regulation of neuronal cell cycle and apoptosis by MicroRNA 34a. Mol Cell Biol.

[42] Olde Loohuis NFM, Kos A, Martens GJM, Van Bokhoven H, Nadif Kasri N, Aschrafi A (2012). MicroRNA networks direct neuronal development and plasticity. Cell Mol Life Sci CMLS.

[43] Ottesen EW (2017). ISS-N1 makes the first FDA-approved drug for spinal muscular atrophy. Transl Neurosci.

[44] Park S-Y, Ferreira A (2005). The generation of a 17 kDa neurotoxic fragment: an alternative mechanism by which tau mediates beta-amyloid-induced neurodegeneration. J Neurosci Off J Soc Neurosci.

[45] Patrick E, Rajagopal S, Wong H-KA, McCabe C, Xu J, Tang A (2017). Dissecting the role of non-coding RNAs in the accumulation of amyloid and tau neuropathologies in Alzheimer’s disease. Mol Neurodegener.

[46] Pichler S, Gu W, Hartl D, Gasparoni G, Leidinger P, Keller A (2017). The miRNome of Alzheimer’s disease: consistent downregulation of the miR-132/212 cluster. Neurobiol Aging.

[47] Pooler AM, Phillips EC, Lau DHW, Noble W, Hanger DP (2013). Physiological release of endogenous tau is stimulated by neuronal activity. EMBO Rep.

[48] Rao MV, McBrayer MK, Campbell J, Kumar A, Hashim A, Sershen H (2014). Specific calpain inhibition by calpastatin prevents tauopathy and neurodegeneration and restores normal lifespan in tau P301L mice. J Neurosci Off J Soc Neurosci.

[49] Rapoport M, Dawson HN, Binder LI, Vitek MP, Ferreira A (2002). Tau is essential to beta-amyloid-induced neurotoxicity. Proc Natl Acad Sci USA.

[50] Reinecke JB, DeVos SL, McGrath JP, Shepard AM, Goncharoff DK, Tait DN (2011). Implicating calpain in tau-mediated toxicity in vivo. PLoS One.

[51] Rissman RA, Poon WW, Blurton-Jones M, Oddo S, Torp R, Vitek MP (2004). Caspase-cleavage of tau is an early event in Alzheimer disease tangle pathology. J Clin Investig.

[52] Salta E, De Strooper B (2017). microRNA-132: a key noncoding RNA operating in the cellular phase of Alzheimer’s disease. FASEB J.

[53] Salta E, Sierksma A, Vanden Eynden E, De Strooper B (2016). miR-132 loss de-represses ITPKB and aggravates amyloid and TAU pathology in Alzheimer’s brain. EMBO Mol Med.

[54] Schaefer A, O’Carroll D, Tan CL, Hillman D, Sugimori M, Llinas R, Greengard P (2007). Cerebellar neurodegeneration in the absence of microRNAs. J Exp Med.

[55] Smith PY, Delay C, Girard J, Papon M-A, Planel E, Sergeant N (2011). MicroRNA-132 loss is associated with tau exon 10 inclusion in progressive supranuclear palsy. Hum Mol Genet.

[56] Smith PY, Hernandez-Rapp J, Jolivette F, Lecours C, Bisht K, Goupil C (2015). miR-132/212 deficiency impairs tau metabolism and promotes pathological aggregation in vivo. Hum Mol Genet.

[57] van Spronsen M, van Battum EY, Kuijpers M, Vangoor VR, Rietman ML, Pothof J (2013). Developmental and activity-dependent miRNA expression profiling in primary hippocampal neuron cultures. PLoS One.

[58] Takashima A, Noguchi K, Sato K, Hoshino T, Imahori K (1993). Tau protein kinase I is essential for amyloid beta-protein-induced neurotoxicity. Proc Natl Acad Sci USA.

[59] Takeuchi H, Iba M, Inoue H, Higuchi M, Takao K, Tsukita K (2011). P301S mutant human tau transgenic mice manifest early symptoms of human tauopathies with dementia and altered sensorimotor gating. PLoS One.

[60] Valor LM, Viosca J, Lopez-Atalaya JP, Barco A (2013). Lysine acetyltransferases CBP and p300 as therapeutic targets in cognitive and neurodegenerative disorders. Curr Pharm Des.

[61] Wagshal D, Sankaranarayanan S, Guss V, Hall T, Berisha F, Lobach I (2015). Divergent CSF τ alterations in two common tauopathies: Alzheimer’s disease and progressive supranuclear palsy. J Neurol Neurosurg Psychiatry.

[62] Walsh DM, Thulin E, Minogue AM, Gustavsson N, Pang E, Teplow DB, Linse S (2009). A facile method for expression and purification of the Alzheimer’s disease-associated amyloid beta-peptide. FEBS J.

[63] Wanet A, Tacheny A, Arnould T, Renard P (2012). miR-212/132 expression and functions: within and beyond the neuronal compartment. Nucleic Acids Res.

[64] Wang Y, Veremeyko T, Wong AH-K, El Fatimy R, Wei Z, Cai W, Krichevsky AM (2017). Downregulation of miR-132/212 impairs *S*-nitrosylation balance and induces tau phosphorylation in Alzheimer’s disease. Neurobiol Aging.

[65] Wayman GA, Davare M, Ando H, Fortin D, Varlamova O, Cheng H-YM (2008). An activity-regulated microRNA controls dendritic plasticity by down-regulating p250GAP. Proc Natl Acad Sci USA.

[66] Wei Z, Batagov AO, Schinelli S, Wang J, Wang Y, EL Fatimy R (2017). Coding and noncoding landscape of extracellular RNA released by human glioma stem cells. Nat Commun.

[67] Wong H-KA, Veremeyko T, Patel N, Lemere CA, Walsh DM, Esau C (2013). De-repression of FOXO3a death axis by microRNA-132 and -212 causes neuronal apoptosis in Alzheimer’s disease. Hum Mol Genet.

[68] Yoshiyama Y, Higuchi M, Zhang B, Huang S-M, Iwata N, Saido TC (2007). Synapse loss and microglial activation precede tangles in a P301S tauopathy mouse model. Neuron.

[69] Zhao X, Kotilinek LA, Smith B, Hlynialuk C, Zahs K, Ramsden M (2016). Caspase-2 cleavage of tau reversibly impairs memory. Nat Med.

[70] (2018) Ionis Pharmaceuticals Initiates Clinical Study of IONIS-MAPT Rx in patients with Alzheimer’s disease. Ionis Pharm. Inc., Carlsbad. http://ir.ionispharma.com/news-releases/news-release-details/ionis-pharmaceuticals-initiates-clinical-study-ionis-mapt-rx. Accessed 13 Oct 2017

