# Supplementary material for: MicroRNA-132 provides neuroprotection for tauopathies via multiple signaling pathways
Source: Acta Neuropathol. 2018 Jul 7;136(4):537–55. doi: 10.1007/s00401-018-1880-5 (PMC6132948; doi:10.1007/s00401-018-1880-5)
Supplement: Supplementary file 1 — Supplementary material 1 (PDF 1251 kb) [file 401_2018_1880_MOESM1_ESM.pdf]

Electronic Supplementary Material

MicroRNA-132 provides neuroprotection for tauopathies via multiple signaling pathways

Rachid El Fatimy[1*], Shaomin Li[1], Zhicheng Chen[1], Tasnim Mushannen[1], Sree Gongala[1], Zhiyun Wei[1], Darrick T. Balu[2], Rosalia Rabinovsky[1], Adam Cantlon[1], Abdallah Elkhal[3], Dennis J. Selkoe[1], Kai C. Sonntag[2], Dominic M. Walsh[1], Anna M. Krichevsky[1,4*]

1. Ann Romney Center for Neurologic Diseases, Department of Neurology, Brigham and Women's Hospital and Harvard Medical School, Boston, MA, 02115, USA.
2. Department of Psychiatry, McLean Hospital and Harvard Medical School, Belmont, MA, 02478, USA
3. Division of Transplant Surgery and Transplantation Surgery Research Laboratory, Brigham and Women's Hospital and Harvard Medical School, Boston, MA, USA.
4. Harvard Medical School Initiative for RNA Medicine, Boston, MA, 02115, USA.

* Corresponding authors:

Anna M. Krichevsky
Ann Romney Center for Neurologic Diseases
Department of Neurology,
Brigham and Women's Hospital,
60 Fenwood Rd, 9006, Boston, MA 02115, USA.
Email: akrichevsky@bwh.harvard.edu

Rachid EL Fatimy
Ann Romney Center for Neurologic Diseases
Department of Neurology,
Brigham and Women's Hospital,
60 Fenwood Rd, 9006, Boston, MA 02115, USA.
Email: relfatimy@bwh.harvard.edu

Legends for Supplemental Figures

***Figure S1*. Validation of miRNA inhibitors, generation of partly aggregated Aβ, and schematics indicating the time course of neuroprotection experiments.** (**a**) Mouse primary neurons were transfected with LNA-containing oligonucleotide inhibitors for the indicated miRNAs, and their effects on validated direct mRNA targets [7] examined 48 hours later by qRT-PCR analysis**.** *P<0.05, **P<0.01 n=3, student t-test. **(b)** Aggregation of SEC-isolated Aβ (1– 42) was followed using a continuous thioflavin T (ThT)-binding assay and fluorescence values are expressed in relative fluorescence units and plotted versus time. The point at which sample was collected (1⁄2*t* max) and used for toxicity experiments is indicated with the blue oval. Freshly SEC-isolated, unaggregated monomer (i.e. equivalent to t=0) was used as a control. **(c)** Time course of neuroprotection experiments on mouse and human primary neurons exposed to Aβ or **(d)** excitotoxic glutamate. **(e)** Spearman rank for the correlation between miRNA levels in primary mouse cortical and hippocampal neurons at DIV-21 and their level of neuroprotection against Aβ toxicity exhibited in Figure 1a. The meta-analysis of miRNA expression was performed on the reported RNAseq and array-based datasets [57, 66].

***Figure S2.* MiR-132 directly targets Tau modifiers RBfox1, GSK3β, EP300, and Calpain 2.** (a) Quantification of three independent Western blot experiments shown in Fig. 4b indicates that miR-132 mimic reduces the levels of GSK3β, RBfox1, and EP300 proteins in primary mouse neurons. **(b)** Quantification of three independent Western blot experiments shown in Fig. 4f indicates that RBfox1 silencing reduces the levels of total Tau protein in primary neurons. **(c)** Quantification of three independent Western blot experiments shown in Fig.4h demonstrate that miR-132 reduces the levels of calpain 2 and cleaved caspase-3 and caspase-7. *P<0.05, **P<0.01 n=3, student t-test.

***Figure S3.* miR-132 reduces Gsk3β activity in mouse primary neurons.** GSK-3β was immunoprecipitated from mouse neurons transfected with miR-132 or control oligonucleotide, and its activity monitored using Tau-S396 as a substrate in an ELISA Assay (phosphoELISA™ Kit from

Invitrogen). miR-132 reduced GSK-3β-mediated phosphorylation of Tau, compared with the scrambled control oligonucleotide. *P<0.02.

***Figure S4*. Mapping of the RBfox1- Tau mRNA interaction in mouse primary neurons. (a)** Schematic presentation of the iCLIP experimental workflow. **(b)** RNA immunoprecipitated with the RBfox1-specific antibody was analyzed by qRT-PCR with primers amplifying various regions of Tau mRNA, including the coding region and 3'UTR. The data are presented as fold enrichment relative to IgG control; mean +/- SEM, n=3, *P<0.005. (**c**) Schematic view of the putative Rbfox1-binding GCAUG motifs in Tau mRNA. In mouse neurons, Rbfox1 binds to the Tau mRNA preferentially via the GCAUG site found in the coding region.

***Figure S5*. Schematic diagram of constructs used for the lentivirus production of LV-miR132 and the corresponding negative control (Empty vector).**

***Figure S6*. Representative images of PHF1-Tau staining in WT and PS19 brain sections, at 7.5 months.** Scale bar=100μm.

***Figure S7*. Stereotactic injections of the LV-miR132 to the CA1 reduces hippocampal atrophy in the PS19 mice. (a)** DAPI staining of PS19 mouse hippocampal CA1 region, injected with the LV-miR132 or control, **(b)** Image J quantification of the hippocampal area (mm2), in the brains injected with either LV-miR132 or control virus. For all quantifications, n = 14 mice, 15 sections per brain. All graphical data are shown as mean +/- SEM, Student's *t*-Tests, *P<0.01.

***Figure S8*. Early supplementation of miR-132 prevents neuronal loss and Tau pathology in PS19 mice. (a)** Timeline of LV-miR132 injections to young PS19 mice and brain analysis. **(b)** Image J quantification of cells positive for cleaved caspase-3, NeuN, PHF-Tau and GFAP in CA1 and adjacent cortical layers. For all quantifications, n = 7 mice, 15 sections per brain. All graphical data are shown as mean +/- SEM, Student's *t*-Tests, *P<0.005.

Supplementary Figure 1

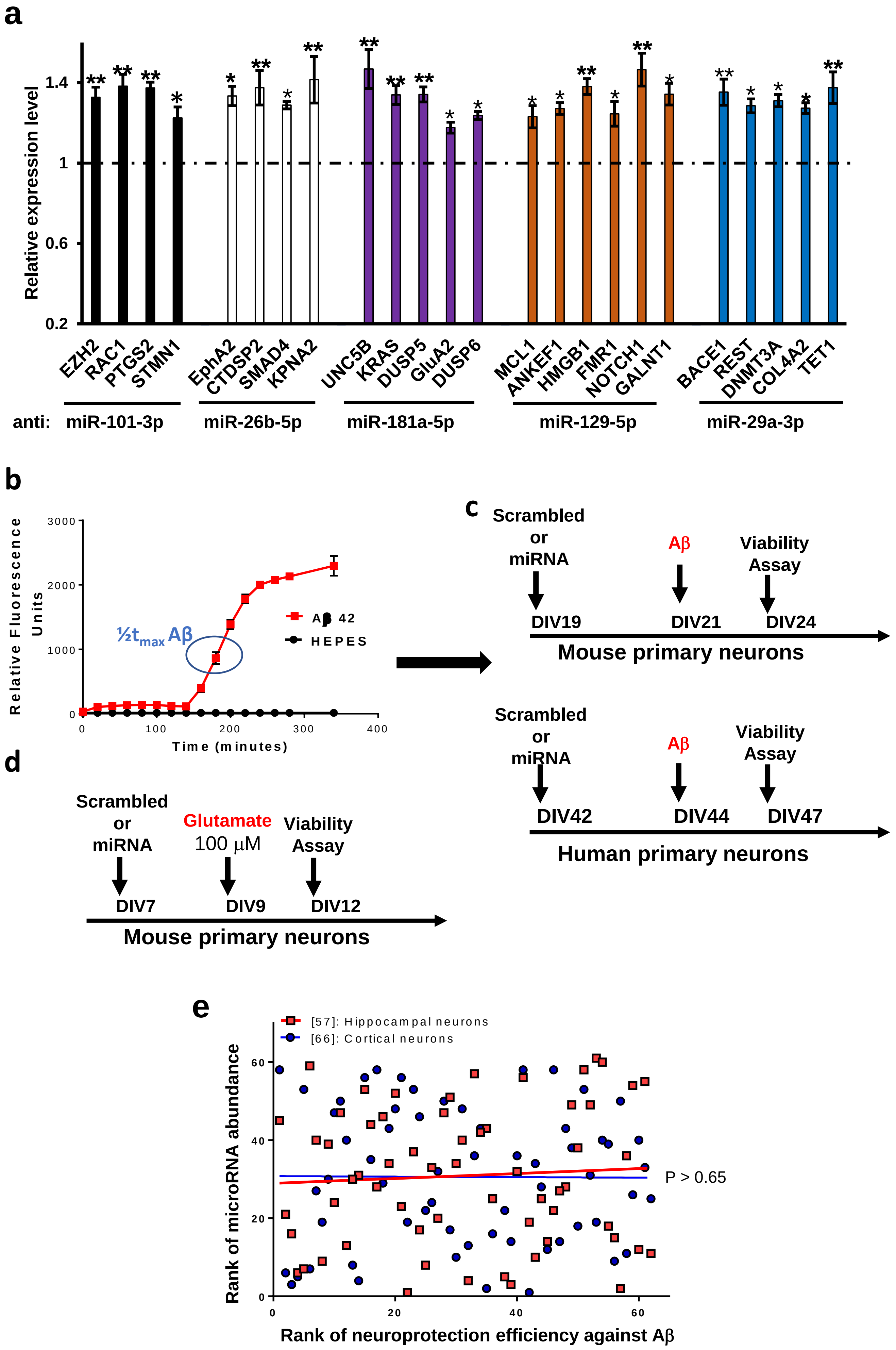

Supplementary Figure 2

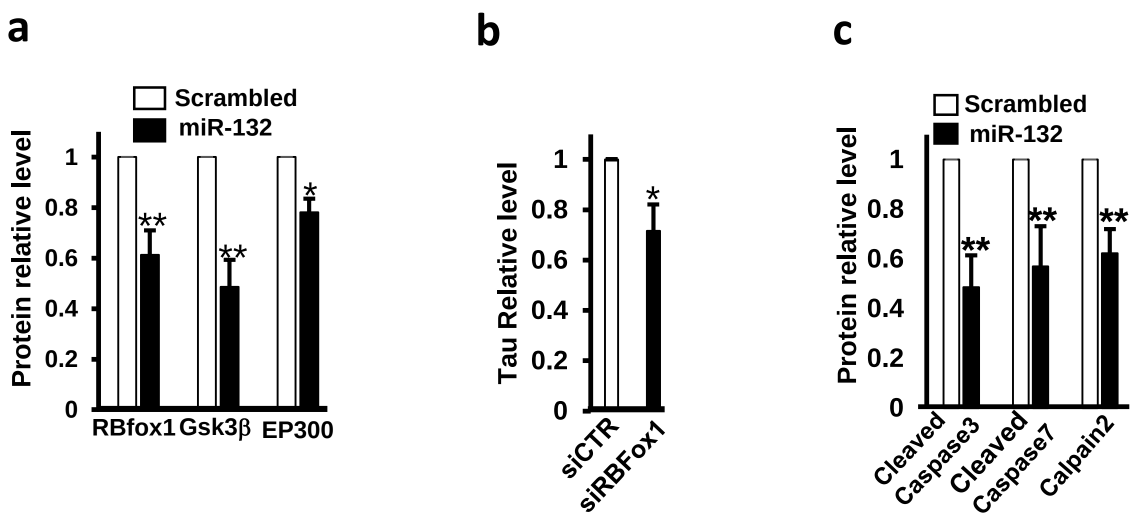

Supplementary Figure 3

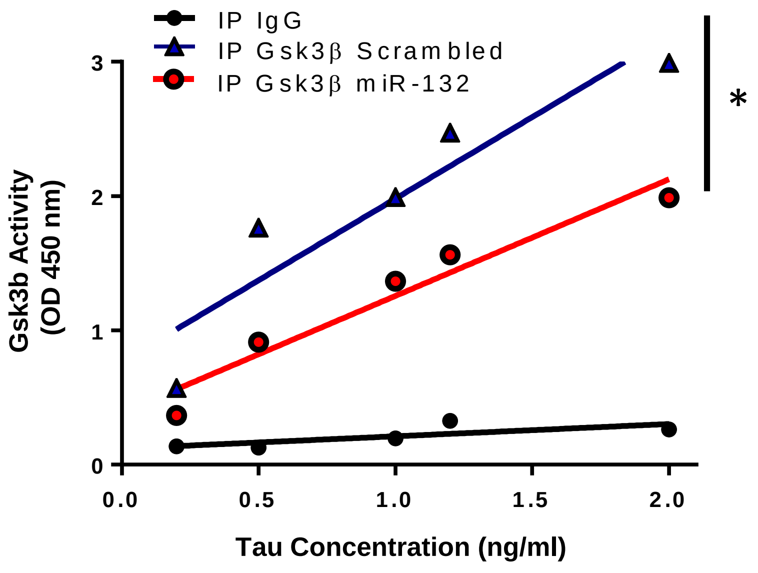

Supplementary Figure 4

a

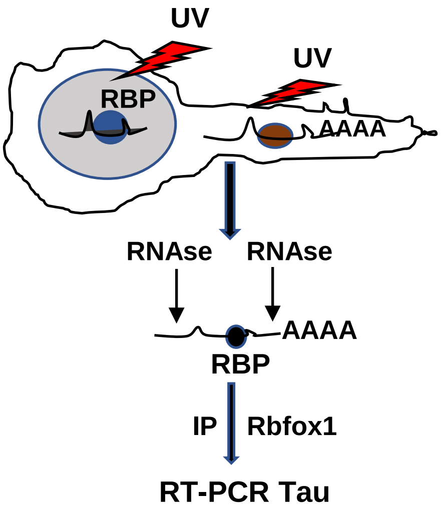

b

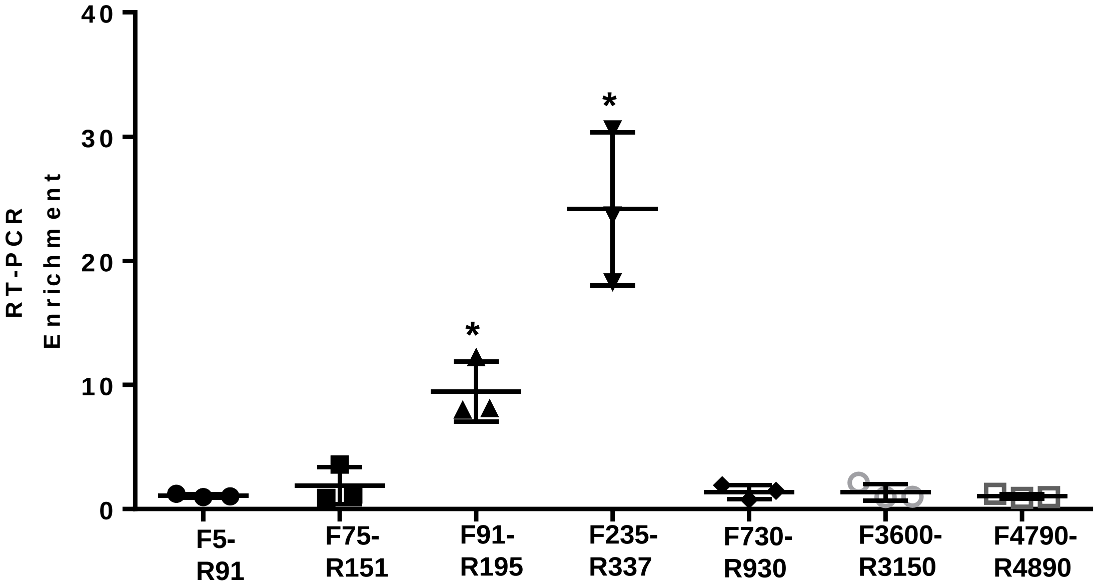

c

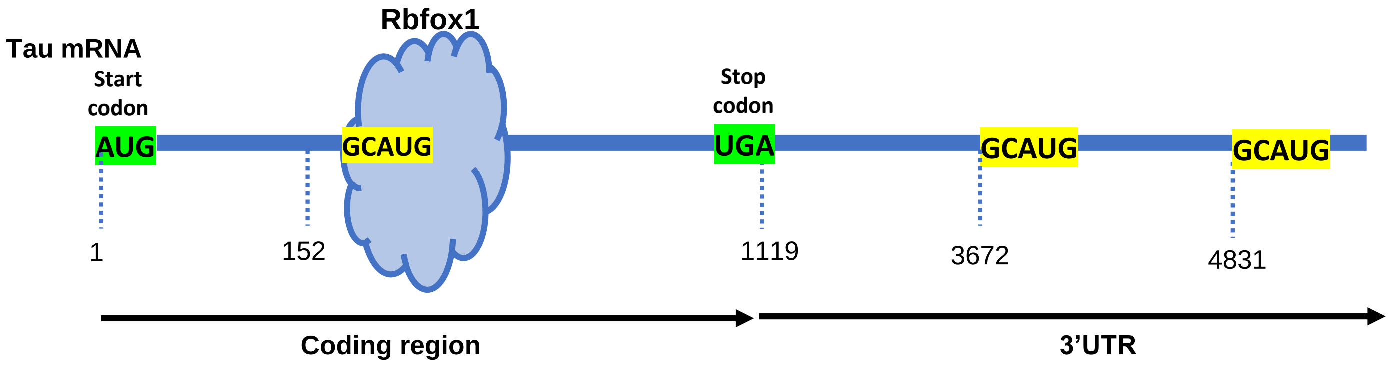

Supplementary Figure 5

miRNA-132 vector: PL13-pSyn-mmu-miR-132-IRES2-EGFP

Empty vector: PL13-pSyn-IRES2-EGFP

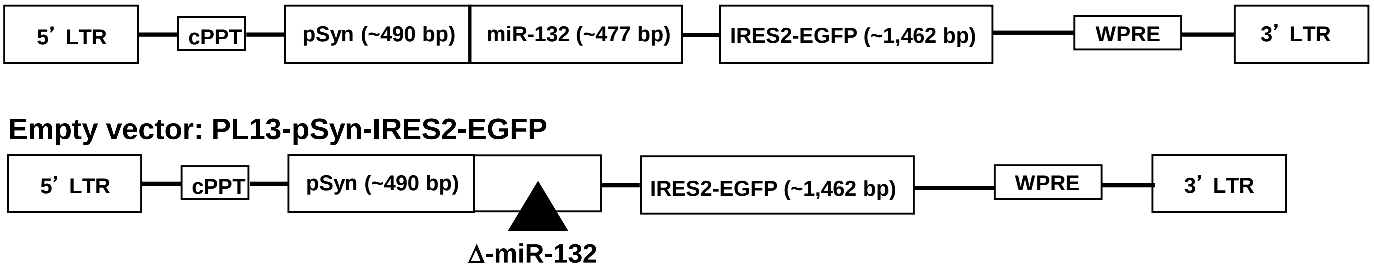

Supplementary Figure 6

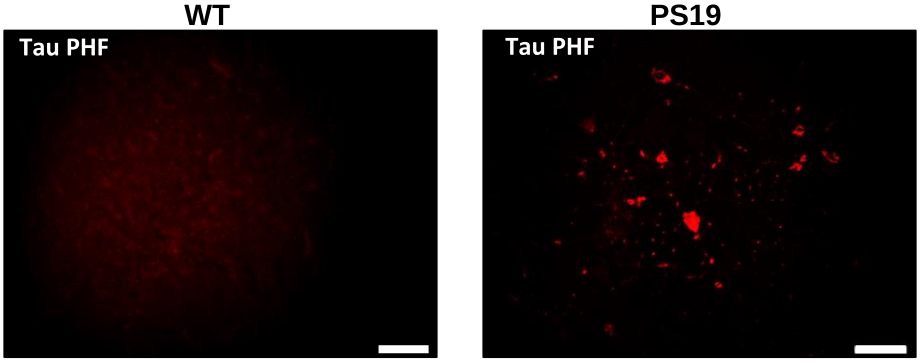

Supplementary Figure 7

a

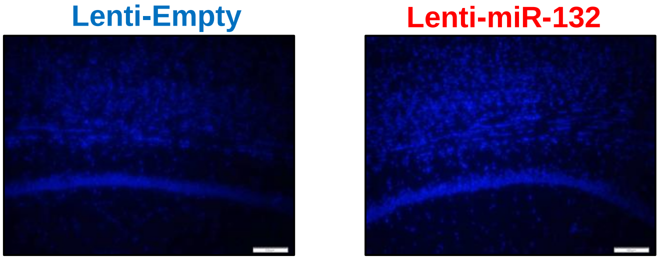

b

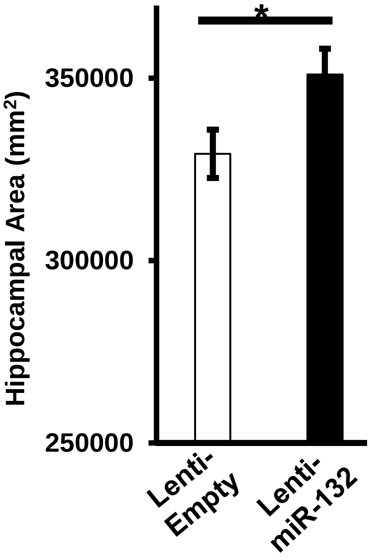

Supplementary Figure 8

a

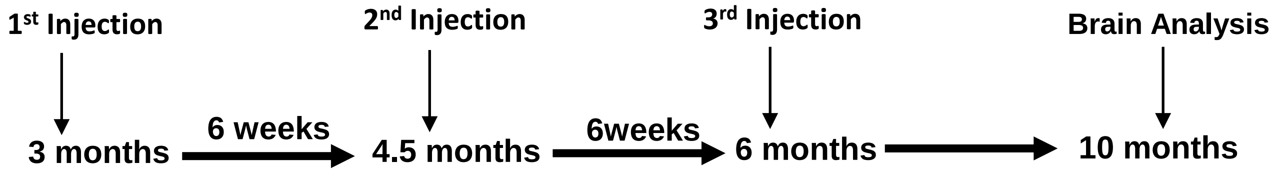

b

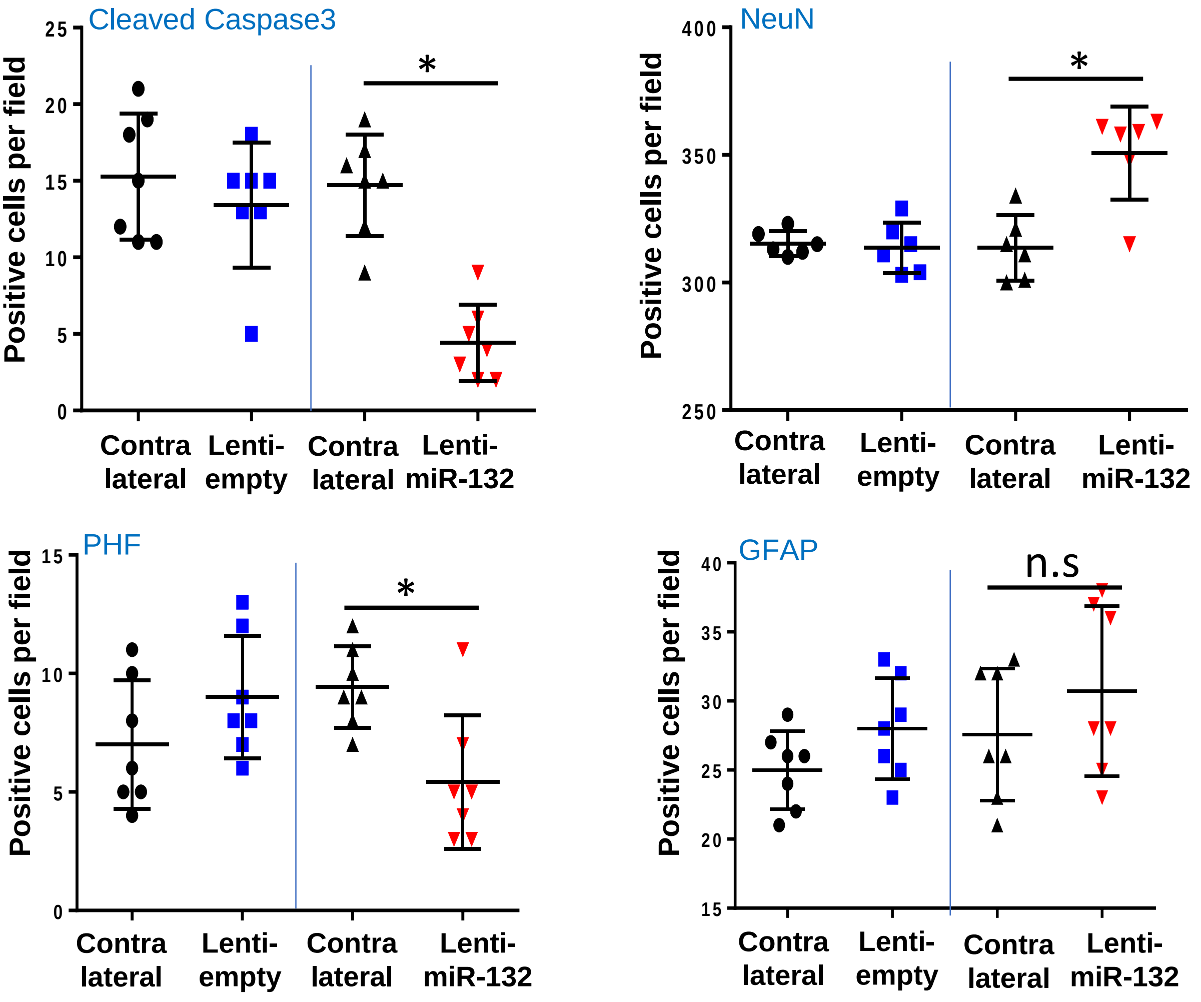

Table S1: List of primers used for real-time PCR experiments

Gene	Accession No.	Primer sequence 5' to 3'	
ACTB	NM_001101.3	F	CACCTTCTACAATGAGCTGCGTGTG
		R	ATAGCACAGCCTGGATAGCAACGTAC
18S rRNA	NR_003286.2	F	ACCACATCCAAGGAAGGCAG
		R	CCGCTCCCAAGATCCAACTA
PABP2	NM_004643.3	F	CAGTTGGCGTGAAGAGAGGA
		R	AGTACACGAGAAGGAGCACC
FOXO3a	NM_001455.3	F	GGCAAAGCAGACCCTCAAAC
		R	TGAGAGCAGATTTGGCAAAGG
Calpain 2	NM_009794.3	F	GAGGTCCTCAACCGCTTCAA
		R	AGCTTGCCACTCCCATCTTC
GSK3β	NM_001347232.1	F	AGCCTTCAGCTTTTGGTAGCAT
		R	CTGCTCCTGGTGAGTCCTTT
EP300	NM_177821.6	F	CTATGGGCTATGGACCTCGC
		R	TGAGCTTGTTGAGGCAGAGTAG
Rbfox1	NM_001359724.1	F	TCTTATGGCGTGCCCATGAT
		R	ACCGGAAAGGGATGTTGGAC
Tau F5-R91	NM_001038609.2	R	ACCAGTATGGCTGACCCT
		F	TCGCCAGGAGTTTGACACAA
Tau F75-R151	NM_001038609.2	R	TCTTGGTCTTGGAGCAGAGTG
		F	GAACCAGTATGGCTGACCCTC
Tau F91-R195	NM_001038609.2	R	ATGCCTGCTTCTTCGGCTTT
		F	CCGAGGTGTGGCGATCTTC
Tau F235-R337	NM_001038609.2	R	TGTGACTCAAGCTCGTGTGG
		F	CACCCGGGACGTGTTTGATA
Tau F730-R930	NM_001038609.2	R	TCTGCAGGCGGCTCTTACTA
		F	CATCACGTGGTGGGCTAGAA
Tau F3600-R3150	NM_001038609.2	R	TCTTCGCCCTGTTACGTTGT
		F	GCTGATTTGTGTCCCTCCCC
Tau F4790-R4890	NM_001038609.2	R	TGAGGGTGGAGGTGGTAATCA
		F	TCTTCGCCCTGTTACGTTGT
Bim	NM_207680	R	GGCGGACAATGTAACGTAACA
		F	CGGAGACGAGTTTAACGCTTA
